# Synthesis, Structural Characterization, and DFT Studies of Fe(III), Co(II), and Ni(II) Mixed‐Ligand Complexes With Albendazole and Nifuroxazide With Molecular Docking Against Respiratory Pathogen Targets

**DOI:** 10.1155/bca/8879576

**Published:** 2026-06-19

**Authors:** Yousef Aldabayan S., Hany M. Abd El-Lateef, Mai M. Khalaf, Aly Abdou

**Affiliations:** ^1^ Department of Respiratory Care, King Faisal University, Al-Ahsa, 31982, Saudi Arabia, kfu.edu.sa; ^2^ Department of Chemistry, College of Science, King Faisal University, Al-Ahsa, 31982, Saudi Arabia, kfu.edu.sa; ^3^ Department of Chemistry, Faculty of Science, Sohag University, Sohag, 82524, Egypt, sohag-univ.edu.eg

**Keywords:** anti-inflammatory, complexes, DFT, DNA gyrase inhibition, molecular docking

## Abstract

In this study, the structural, electronic, and biological characteristics of newly synthesized transition metal complexes FeABNF, CoABNF, and NiABNF, resulting from the chelation compounds albendazole (AB) and nifuroxazide (NF), were studied. The synthesized compounds were found to have excellent yields with high percentage yields (∼80%). Thermal analysis showed that these complexes were highly stable with decomposition temperatures above 300°C. Molar conductivity tests revealed that FeABNF is a 1:1 electrolyte with a value of 38.95 Ω^−1^·cm^2^·mol^−1^, NiABNF is a 1:2 electrolyte with a value of 88.17 Ω^−1^·cm^2^·mol^−1^, while CoABNF is a nonelectrolyte with a value of 9.86 Ω^−1^·cm^2^·mol^−1^. FT‐IR analysis proved that these complexes are bidentate due to their N and O donor atoms. The electronic spectra and magnetic moments confirmed that these compounds had octahedral geometry. The DFT calculation and biological assays showed that the complexes exhibited enhanced antimicrobial activity compared to the free ligands. The investigated compounds exhibited variable antibacterial and antifungal activities, with the metal complexes generally showing enhanced activity compared to the free ligands. However, the degree of activity was found to depend on the nature of the metal ion and the tested microbial strain. The most potent antibacterial action was exhibited by NiABNF and CoABNF complexes, which exhibited 28–30‐mm inhibition zones and 91%–94% activity index, and good antifungal activity against *Candida albicans*, 20 mm. Anti‐inflammatory activity order was determined as the NiABNF complex with IC_50_: 56.97 μM and 93% inhibition. These findings were supported at the molecular docking level, as the NiABNF complex had the highest binding affinity to DNA gyrase B (PDB: 4DUH, −8.90 kcal/mol) and SARS‐CoV‐2 main protease (6LU7, −9.40 kcal/mol) via hydrogen bonding and hydrophobic interactions, which make it a promising therapeutic agent. Both experimental and computational studies reported the same order of bioactivity: NiABNF > CoABNF > FeABNF > free ligands.

## 1. Introduction

Antimicrobial resistance, however, insidiously weakens many treatments. Bacteria employ various tricks to ride out conventional drugs, such as efflux pumps, enzyme inactivation, and genetic changes [1–3]. This has created an urgent push for new compounds that can dodge or beat these resistance strategies [3–5]. Perhaps, one of the most promising ways forward involves using metal coordination chemistry to tweak and boost existing medications [6–11].

Nifuroxazide, belonging to the nitrofuran series, represents a substance with a wide range of antimicrobial activity [12, 13]. It acts by inhibiting dehydrogenases and hence disturbs energy metabolism in bacteria [14, 15]. When nifuroxazide coordinates with transition metals such as Fe(III), Co(II), or Ni(II), the formed metal complexes usually exhibit higher biological activity [16]. Such complexes usually exhibit better solubility, better cellular uptake, and higher bioavailability, which means higher antimicrobial and anticancer effects and anti‐inflammatory activity. Coordination via its nitro and amide groups helps to stabilize the metal center and therefore enhances interaction with biological targets.

Albendazole (AB), as a benzimidazole derivative, has already been demonstrated to effectively combat parasitic diseases through the inhibition of microtubule assembly [17, 18]. Aside from being an anticancer and antimicrobial agent, the compound has also exhibited considerable potency when it binds with Fe(III), Co(II), and Ni(II) [17]. This has enabled the metal complexes to improve their interactions and capability to target micro‐organisms by binding with nucleic acids and important enzymes [19, 20].

Attaching two biologically active ligands to a metal complex, a new concept in medicine, may also provide a solution to drug resistance in microbes [21–23]. This is because a compound like this may have several modes of action, therefore showing certain levels of synergism. A central core formed by a transition element can make two ligands able to interact with a variety of targets. It can also show a more favorable profile, especially compared to other drug therapies, in its ability to deal with drug resistance [21–23].

Mixed‐ligand complexes constitute an important class of coordination compounds in which more than one type of ligand is coordinated to the same metal center [21]. Such systems have attracted considerable attention due to their enhanced structural diversity and tunable physicochemical properties compared to single‐ligand complexes. The presence of different donor environments around the metal ion allows fine control over geometry, electronic distribution, and stability, which can significantly influence the reactivity leading to improved biological, catalytic, optical, and electrochemical properties [21]. Accordingly, the design of mixed‐ligand coordination compounds represents a powerful strategy for developing multifunctional materials with enhanced performance in both chemical and biomedical fields.

Respiratory infections due to bacterial, fungal, and viral pathogens do pose a significant health problem worldwide, particularly to people with weakened immunity, the elderly, and people already suffering from pre‐existing diseases of the respiratory system [24–26]. Common bacterial, fungal, and viral infections of the human respiratory system, as mentioned, include *Klebsiella pneumoniae*, *Staphylococcus aureus*, and the fungus *Candida albicans*, which usually appear as hospital or community‐acquired infections [27–29]. *K*. *pneumoniae*, a Gram‐negative bacterium, usually produces hospital‐acquired pneumonia and exhibits high levels of resistance to multiple drugs. *S*. *aureus*, particularly methicillin‐resistant *S. aureus* (MRSA), is a Gram‐positive bacterial infection that causes a broad range of respiratory infections [30–32]. The fungus *C*. *albicans*, which is harmless to the human body, usually overgrows in weakened immunity and invades the human respiratory tract, leading to life‐threatening candidiasis [33–35]. What even increases the danger of life‐threatening diseases due to these causative agents is the direct effect of the newly emerging SARS‐CoV‐2, the causative agent of COVID‐19, which can immediately affect the lungs, including the mild symptoms of breathing difficulties to severe pneumonia, which can cause the life‐threatening disease acute respiratory distress syndrome [36–38]. The key enzyme that causes the replication of the SARS‐CoV‐2 virus is the main protease (Mpro), also known as 3CLpro, which cleaves the viral polyproteins into constituent peptides, and the SARS‐CoV‐2 Mpro, in particular, despite sharing little sequence homology, possesses significant homology to the overall structure of protease enzymes and can, therefore, serve as the basis of drug design [39–41].

Given the rising problem of resistance of respiratory pathogens to currently deployed drugs, the present study is part of a pressing quest that seeks to unveil new forms of therapy. The project’s main focus is on the evaluation of newly engineered bioactive ligands and metal complexes’ ability to cope with the most dominant respiratory pathogens, *K. pneumoniae*, *S. aureus*, and *Candida albicans*, through an *in vitro* study. Furthermore, it also studies the capability of the ligands to cope with the rising threat of the SARS‐CoV‐2’s Mpro through computer‐based studies. Metal‐based compounds are of significant interest, mainly because of the substantial, broader scope of activity that is often exhibited by them. These compounds are the result of the ability of metals to coordinate with the ligands. As a result, the ligands are better suited to dealing with many biological targets as opposed to their noncomplexed forms. Hence, these compounds are of significant interest and are included as an integral part of the study. It is through a docking study that the ability of the ligands to bind to Mpro is evaluated. Furthermore, the effectiveness of the ligands is also a core component of the research, specifically against the most dominant forms of bacteria and fungi that are difficult to cope with. In essence, the study is focused on the construction of a novel set of ligands made up of a combination of nifuroxazide, AB, Fe(III), Co(II), and Ni(II). Evaluation is of the compounds’ ability to address a range of antibacterial and antifungal, as well as anti‐inflammatory effects.

## 2. Methodology

### 2.1. Reagents and Synthesis

**SCHEME 1:**
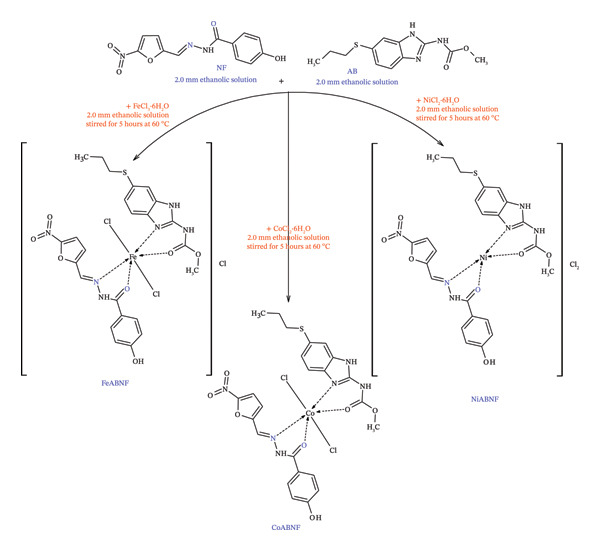
Synthesis of FeABNF, CoABNF, and NiABNF.

All chemical reagents, including the ligands AB and NF, and the metal salts (FeCl_3_·6H_2_O, CoCl_2_·6H_2_O, and NiCl_2_·6H_2_O), were purchased from Sigma‐Aldrich and were of analytical grade (98% purity). The mixed‐ligand complexes were synthesized in a strict 1:1:1 stoichiometric ratio [42]. Briefly, 2.0 mn ethanolic solutions of the metal salts were stirred for 30 min at room temperature. Then, 2.0 mm ethanolic solutions of AB and NF were added dropwise. The reaction mixture was stirred for 5 h at 60°C to ensure complete complexation. The resulting precipitates were filtered, washed with cold ethanol, and recrystallized from hot ethanol, as shown in Scheme 1.

### 2.2. Characterization Techniques

The characterization techniques are listed in detail in supporting data.

### 2.3. DFT Calculation

The study performed theoretical calculations to investigate the ligands and their metal complexes using the program package ORCA 5.0 [43]. To obtain the optimized structures, the calculations were carried out applying density functional theory (DFT) using the B3LYP technique combined with the def2‐SVP basis set for carbon, hydrogen, nitrogen, and oxygen, and the def2‐TZVP basis set for the central metal atom [44–46]. To obtain the atomic coordinates, we used the program package Avogadro, improved by the application of the program package ORCA [47]. After the calculations for optimizing the structures of the ligands, FMOs were computed. Subsequently, the results were analyzed using the program package Multiwfn from the outputs of the program package ORCA [48]. Additionally, the study of the global reactivity descriptors, which include EA, IP, Δ*E*, *χ*, CP, *η*, *σ*, *ω*, and Nu, was performed [49–51].

### 2.4. Biological Activity

The *in vitro* antimicrobial screening, performed using the disc diffusion method, revealed that several of the investigated compounds exhibited noticeable antibacterial activity against both Gram‐positive bacterial strains (*B*. *subtilis* and *S*. *aureus*) as well as Gram‐negative strains (*E*. *coli* and *K*. *pneumoniae*). The measured inhibition zones indicated that the tested compounds were compared with the standard antibiotic chloramphenicol. Similarly, the antifungal assessment was done against the pathogenic fungi *C*. *albicans* and *A*. *niger* and the activity was compared to the reference drug clotrimazole. The antimicrobial effectiveness was further quantified through the calculation of the activity index (AI%), which provided a comparative evaluation of the compounds relative to the standard drugs. The antimicrobial activity of the investigated ligands and their metal complexes is influenced, to some extent, by the choice of solvent. Solvent, DMSO, was employed to ensure adequate solubility and homogeneous distribution of the tested compounds. While the solvent itself exhibited negligible antimicrobial activity, it plays an essential role in facilitating diffusion through the microbial medium and enhancing cell membrane permeability. Additionally, solvent–solute interactions may affect the stability and availability of the active species in solution.

In addition to antimicrobial screening, the synthesized compounds were evaluated for their anti‐inflammatory potential using the egg albumin denaturation assay. The percentage inhibition values obtained at different concentrations were used to construct dose–response curves, from which the IC_50_ values were determined and compared with the reference drug ibuprofen.

The detailed experimental procedures [52, 53], including inhibition zones, AIs, and calculated IC_50_ values for all tested compounds, are provided in the Supporting Data.

### 2.5. Molecular Docking

Molecular docking analysis will be conducted with a deeper exploration of the behavior of AB, NF, and their FeABNF, CoABNF, and NiABNF complexes to gain more information on how this new series behaves and predict their potential to interact with DNA gyrase B (PDB entry: 4DUH) [54]. DNA gyrase B is a well‐known target that is worthy of further consideration in antibacterial drug discovery because of its prominence in DNA replication and transcription in bacteria. It is a part of a type II topoisomerase enzyme complex that catalyzes ATP hydrolysis to supercoil the bacteria’s DNA, a very vital function for the survival and propagation of bacteria in nature. This enzyme is absent in human biology, making it an attractive target for the development of antibacterial agents with a low likelihood of side effects, particularly those arising from cumulative cytotoxic effects. By using 4DUH for docking analysis, we can determine how these new compounds might interact at the enzyme’s active sites through hydrogen bonding, electrostatics, and hydrophobic amino acids that are crucial for enzyme function.

The PDB ID 6LU7 structure was chosen for the docking study because it depicts the crystal structure of the SARS‐CoV‐2 Mpro, also called Virus Mpro. The structure also depicts the interaction of the viral protease with a ligand, providing a well‐defined active site that is essential for the docking study. The viral protease is a significant target for the replication of the SARS‐CoV‐2 virus. The structure is also valuable for the docking study because the viral protease has no human homolog. The structure is of high resolution at 2.16 Å, which is significant for understanding the precise interaction of the ligand. In addition, the ligand in the structure offers an opportunity to validate the docking study. The validation of the structure is significant for making the results obtained accurate for the prediction of the binding affinities of the inhibitors.

The resulting 3D crystal structures obtained from the RCSB Protein Data Bank (https://www.rcsb.org/) are for DNA gyrase B (PDB ID: 4DUH, resolution = 1.50 Å) and Mpro (PDB ID: 6LU7, resolution = 2.16 Å). The docking protocols were carried out with previously described methods [55]. In this case, all simulations were carried out using AutoDock Vina [56]. The predetermined dimensions of the search box were (20.879, 12.334, 23.800) for 4DUH and (−26.041, 12.602, 59.188) for 6LU7. Binding energies, which are expressed in kcal/mol, were applied to rank all compounds according to their ability to bind to the target protein. The results of these protocols can provide a better understanding of the *in vitro* antibacterial activity in relation to binding affinity as well as protein interaction.

## 3. Results and Discussion

### 3.1. FeABNF, CoABNF, and NiABNF Characterization

#### 3.1.1. Physicochemical Properties

The FeABNF, NiABNF, and CoABNF complexes formed the chelation of two versatile ligands, namely, AB and NF. Full structural and coordination chemistry mapping was attempted using an array of physicochemical techniques. They were prepared in good yields, with a yield of about 80%, displaying good thermal stability, being intact above 300°C, Table (S.1). The pale brown color for FeABNF, light green for NiABNF, and reddish for CoABNF point to different ligand‐field environments at the respective metal centers influenced by geometrical factors as well as electronic configuration.

#### 3.1.2. Molar Conductivity Measurements

The molar conductivity measurements proved to be crucial in understanding the ionic nature of the complexes and their formulations in ethanol, as presented in Table (S.2). FeABNF was found to have a moderate molar conductivity of 38.95 Ω^−1^·cm^2^·mol^−1^, typical of 1:1 type electrolyte. This confirms the ionic nature of the compound [Fe(AB) (NF) (Cl)_2_]Cl, where one chloride ion is ionically dissociated. However, NiABNF was found to have a high molar conductance of 88.17 Ω^−1^·cm^2^·mol^−1^, typical of 1:2 type compounds. This suggests that the compound is of the ionic type [Ni(AB) (NF) (Cl)_2_], where both chloride ions are outside the coordination sphere. Moreover, CoABNF was found to have a very low molar conductivity of 9.86 Ω^−1^·cm^2^·mol^−1^, typical of a nonelectrolyte compound where both chlorides are coordinated to the metal center.

#### 3.1.3. Infrared (FT‐IR) Spectral Data

FT‐IR (Figure (S.1) and Table 1), also identified the nature of the coordination. The primary bands that change during the coordination are the stretching vibrations of the carbonyl groups (C=O) and the azomethine groups (C=N). For the NF ligand, the stretching vibration of the carbonyl groups shows a shift from 1670 cm^−1^ to 1640–1645 cm^−1^, while that of the azomethine groups shows a shift from 1585 cm^−1^ to 1562–1566 cm^−1^. For AB ligand, the stretching vibration of the carbonyl groups changes from 1664 cm^−1^ to 1618–1627 cm^−1^, while that of the azomethine groups changes from 1585 cm^−1^ to 1617–1620 cm^−1^. The shift in these bands showed that electrons are moving from the donor sites toward the metal atom, thus proving that electrons are distributed between the donor sites. Additional evidence indicating coordination between the metal and the donor atoms is given by new bands at 567–572 cm^−1^ (M–O) and at 498–510 cm^−1^ (M–N).

**TABLE 1:** IR of the FeABNF, NiABNF, and CoABNF complexes.

	**NF**	**AB**	**FeABNF**	**NiABNF**	**CoABNF**

υ (–C=O)_NIF_	1670	—	1645	1640	1644
υ (–C=N)_NIF_	1585	—	1562	1566	1564
υ (–C=O)_Alb_	—	1664	1618	1627	1620
υ (–C=N)_Alb_	—	1633	1620	1618	1617
υ (M–O)	—	—	567	558	572
υ (M–N)	—	—	510	498	507

#### 3.1.4. UV–Visible Spectra

**FIGURE 1:**
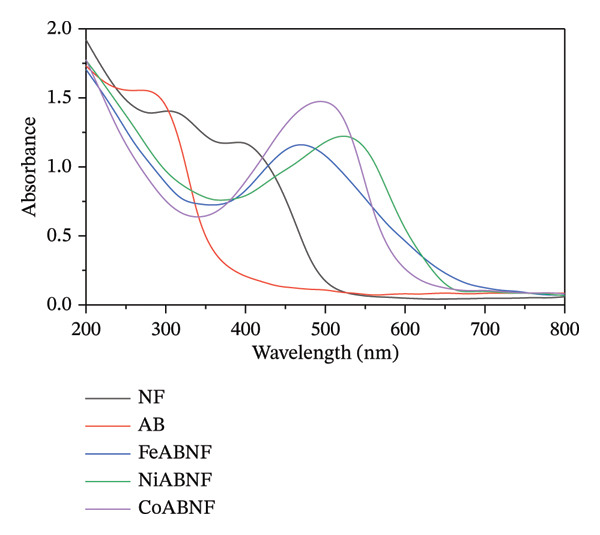
UV–vis spectra of NF, AB, FeABNF, NiABNF, and CoABNF.

Higher insights into the geometry and d–d transitions of the metal ions can be gained from the UV–visible spectral studies, as summarized in Table (S.3) and Figure 1. The spectral profile of the FeABNF complex is indicative of the spin‐allowed ^6^A_1g_(F)⟶^4^E_g_(G) transition arising at 455 nm or 21,978 cm^−1^. This is characteristic of the low‐spin Fe(III) ion in an octahedral environment. In the case of the NiABNF complex, the presence of the absorption band at 545 nm or 18,349 cm^−1^ is ascribed to the spin‐allowed ^3^T_1_(F)⟶^3^T_1_(P) transition expected of the low‐spin, tetrahedral, and Ni(II) ion. For the CoABNF complex, the electronic spectral profile shows the presence of the transition band at 510 nm or 19,608 cm^−1^, corresponding to the ^4^T_1_g(F)⟶^4^T_2_g(P) transition for the low‐spin octahedral Co(II) ion.

#### 3.1.5. Magnetic Moment Values

These magnetic moment results agreed with the structural findings as well (Table (S.4)). For the FeABNF complex, the magnetic moments recorded had just approximately 1.88 Bohr magnetons. These findings account for a low‐spin d^5^ configuration with one unpaired electron, necessary for the octahedral Fe(III) complex. In the case of the NiABNF complex, the magnetic moments had approximately 3.18 Bohr magnetons. These results accounted for a high‐spin configuration with all unpaired electrons necessary for a tetrahedral nickel complex. Finally, the magnetic moment for the CoABNF complex had approximately 1.81 Bohr magnetons, which accordingly represented a low‐spin configuration with one unpaired electron as necessary for octahedral geometry.

#### 3.1.6. Job’s Method of Continuous Variation

Job plot measurements performed by Job’s method show that the stoichiometric ratio of the metal:AB:NF remains constant at 1:1:1 through all the compounds. This is evident from Job plot measurements with a constant stoichiometric ratio of 1:1:1 for the metal:AB:NF, as shown in Figure (S.2). This confirms the postulated compounds [Fe(AB) (NF) (Cl)_2_]Cl, [Ni(AB)(NF)]Cl_2_, and [Co(AB) (NF) (Cl)_2_].

#### 3.1.7. Mass Spectrometric Data

**FIGURE 2:**
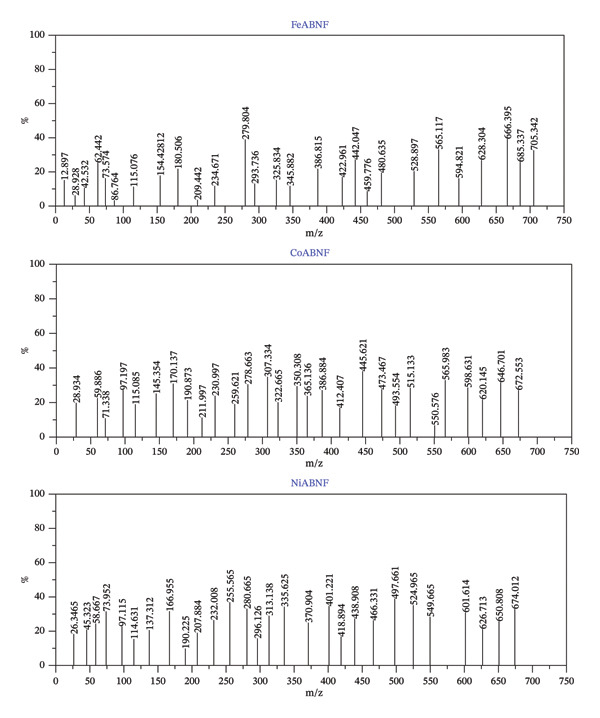
Mass spectra of the FeABNF, NiABNF, and CoABNF complexes.

**SCHEME 2:**
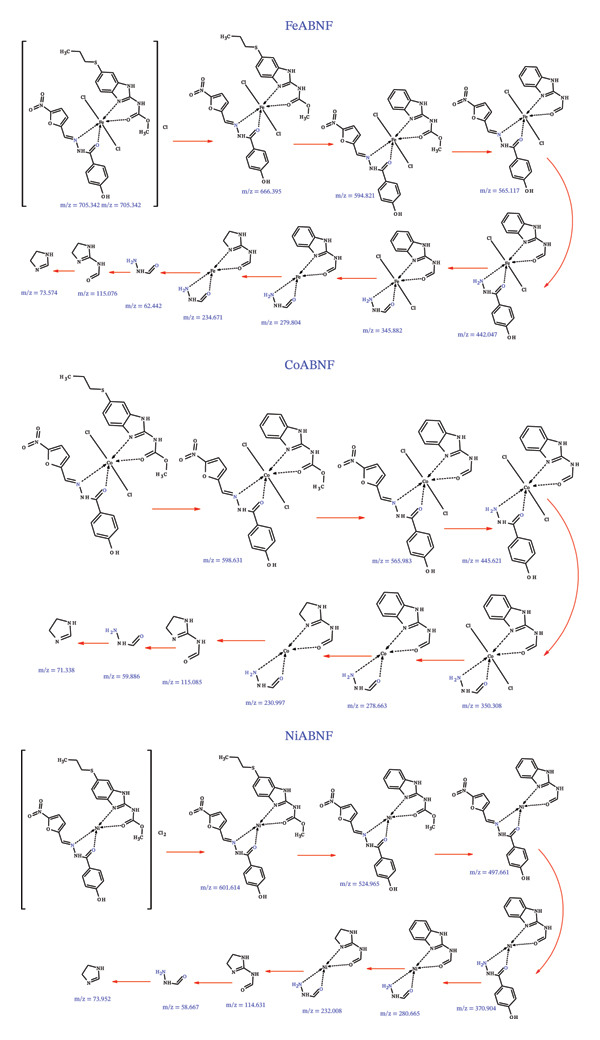
Mass fragmentation of FeABNF, NiABNF, and CoABNF.

Mass spectrometry results also confirm the above structures (Figure 2, Scheme 2). FeABNF has a mass of 705.342 consistent with the calculated molecular formula of FeABNF: C_24_H_24_Cl_3_FeN_6_O_7_S of molecular weight 702.75. NiABNF and CoABNF give mass spectrometry results of 674.012 and 672.553 amu, respectively, consistent with the molecular formula of these compounds: C_24_H_24_Cl_2_N_6_NiO_7_S and C_24_H_24_Cl_2_CoN.

#### 3.1.8. Elemental Analysis (CHN)

CHN analysis showed a good correlation with the obtained figures, as presented in Table 2. In the case of FeABNF, the obtained percentages were 41.98% for C (calc 41.02%), 3.07% for H (calc 3.44%), 12.43% for N (calc 11.96%), and 8.44% for Fe (calc 7.95%). A strong correlation was observed for NiABNF and CoABNF. This proved that the incorporated compounds were within the formulated stoichiometry.

**TABLE 2:** Elemental analysis: found (calculated) of the FeABNF, NiABNF, and CoABNF complexes.

Elements	Complexes		
	FeABNF	NiABNF	CoABNF
C	41.98 (41.02)	43.87 (43.01)	43.67 (43.00)
H	3.07 (3.44)	3.11 (3.61)	3.27 (3.61)
N	12.43 (11.96)	13.24 (12.54)	13.31 (12.54)
M	8.44 (7.95)	8.12 (8.76)	8.19 (8.79)

#### 3.1.9. Thermal Analysis

**FIGURE 3:**
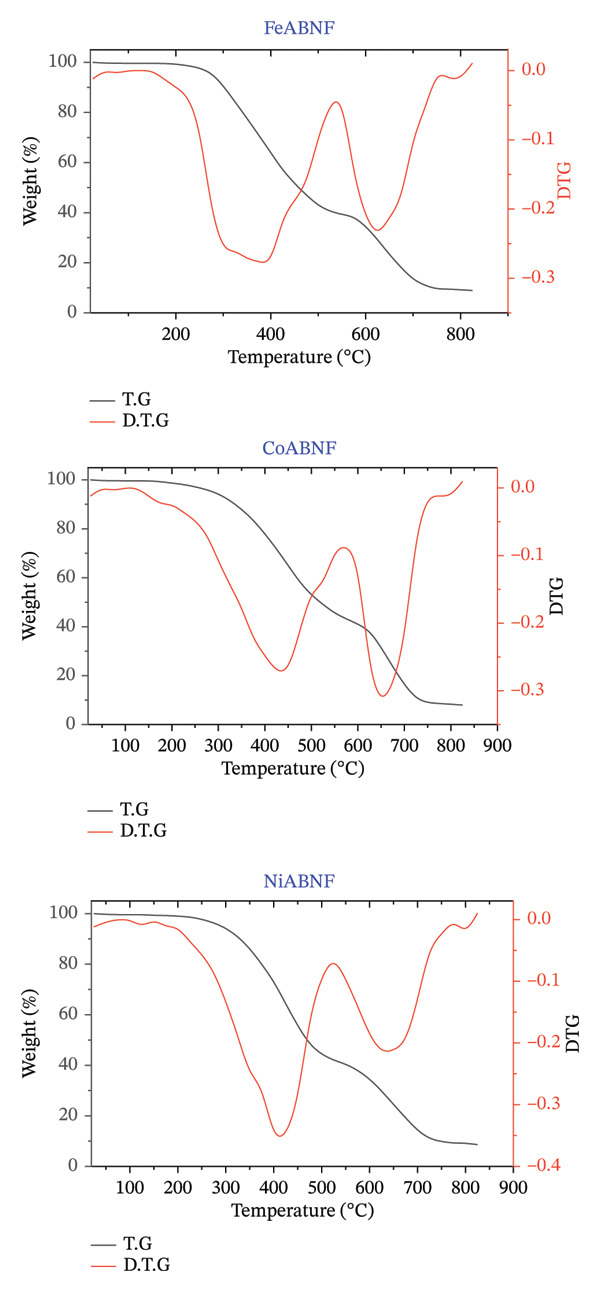
Thermal degradation of the FeABNF, NiABNF, and CoABNF complexes.

The information derived from the thermogravimetric analysis of FeABNF, NiABNF, and CoABNF is given clearly as represented in Table 3 and Figure 3, where all three complexes are seen to decompose in two steps with no loss of mass observed until a temperature of approximately 230°C. This gives insight into there being no water of hydration inclusive in any of the final structures, as water, whether hydration or coordinated, tends to vaporize at lower temperatures than this (hydration water < 150°C; coordinated water < 200°C).

**TABLE 3:** Thermal degradation of FeABNF, NiABNF, and CoABNF.

	**TG (** ^**o**^ **C)**	**DTG (** ^**o**^ **C)**	**Mass loss (%)**		**Residue (%)**	
			**Found (calculated)**	**Assignment**	**Found (calculated)**	**Assignment**

FeABNF	250–545	378	61.067 (61.874)	C_15_H_18_Cl_3_NO_5_S	38.566 (38.671)	C_9_H_6_FeN_5_O_2_
	545–720	620	30.648 (30.771)	C_9_H_6_N_5_O_2_	7.939 (8.145)	Fe
NiABNF	230–560	435	58.646 (59.354)	C_15_H_18_Cl_2_NO_5_S	40.817 (41.008)	C_9_H_6_CoN_5_O_2_
	560–745	650	32.073 (31.542)	C_9_H_6_N_5_O_2_	8.754 (8.681)	Co
CoABNF	255–520	415	58.773 (58.016)	C_15_H_18_Cl_2_NO_5_S	40.870 (40.695)	C_9_H_6_N_5_NiO_2_
	520–725	640	32.143 (33.308)	C_9_H_6_N_5_O_2_	8.773 (8.803)	Ni

Considering FeABNF as an illustration, the temperature range for the first step of decomposition for FeABNF ranges from 250°C to 545°C. The maximum decomposition rate is observed at 378°C, with 61.067% (theoretical 61.874%) mass loss. The degradation of FeABNF at 250°C–545°C leads to the evolution of the fragment C_15_H_18_Cl_3_NO_5_S, resulting in the residual composition of C_9_H_6_FeN_5_O_2_. The temperature interval of the second step of decomposition for FeABNF ranges from 545°C to 720°C. The DTG peak of FeABNF in the second step of degradation occurs at 620°C, with 30.648% (theoretical 30.771%) mass loss. This step of degradation results in the evolution of the fragment C_9_H_6_N_5_O_2_, ultimately yielding elemental iron (Fe) as a residue of 7.939% (calculated 8.145%).

In the case of the NiABNF complex, decomposition occurs slightly earlier, covering a temperature range of approximately 230°C–560°C, with a maximum rate in the DTG curve observed at a temperature of 435°C. It starts with a loss of C_15_H_18_Cl_2_NO_5_S, with a calculated mass loss of 58.646%, compared to a theoretical value of 59.354%. This decomposition leaves a residue of 40.817%, compared to a theoretical calculation of 41.008%, composed of C_9_H_6_CoN_5_O_2_. The second decomposition step, occurring between 560°C and 745°C with a maximum at approximately 650°C, corresponds to a weight loss attributed to the elimination of a C_9_H_6_N_5_O_2_ fragment.

The process by which the CoABNF complex decomposes is a two‐step process that is similar. The first stage of decomposition occurs between 255°C and 520°C, with a DTG peak at 415°C, and involves a weight loss of 58.77% (calculated to be 58.02%), which is corresponding to the removal of the same organic moiety, C_15_H_18_Cl_2_NO_5_S. The residue recorded after this stage is 40.87% (calculated to be 40.70%), which is similarly attributed to C_9_H_6_N_5_NiO_2_. The second decomposition stage takes place between 520°C and 725°C. During this process, the DTG peak is recorded at 640°C. During this process, C_9_H_6_N_5_O_2_ is recorded to be lost (32.14%; calculated to be 33.31%), while elemental nickel, Ni, is the final residue of 8.773% (calculated 8.803%).

That is to say, the TG/DTG curves of the FeABNF, NiABNF, and CoABNF complexes exhibit a consistent thermal degradation mechanism of two steps without any initial weight loss caused by water loss. Moreover, good correlation between experimental and theoretical weight loss is evident.

#### 3.1.10. pH Stability

This is addressed by the pH stability profile for FeABNF, NiABNF, and CoABNF (Figure (S.3)). The stability of the complexes was monitored over a pH range of 2.0–10.0 in an aqueous universal buffer system. All three present a somewhat “bell‐shaped” absorbance pattern, with maximum stability near‐neutral slightly basic conditions of pH ∼6–8, suggesting an optimistic coordination environment around the metal center under near‐physiological pH. At very acid (pH < 4) and very basic (pH > 9) extremes, absorbance drops significantly, which indicates partial dissociation or structural disruption.

The combined evidences obtained from elemental analysis, conductivity, FT‐IR, UV–vis, magnetic susceptibility, and mass spectrometry, together with Job’s method studies, thus supporting that FeABNF and CoABNF are low‐spin octahedral complexes containing two chloride ligands terminally bonded to the metal. By contrast, NiABNF has a tetrahedral geometry with two chloride counterions being present outside the coordination sphere. Both AB and NF act as the bidentate N,O donor ligand, chelating through azomethine nitrogen and carbonyl oxygen to lock the metal into predictable, well‐defined coordination environment as depicted in Scheme 1.

### 3.2. DFT Calculations

**FIGURE 4:**
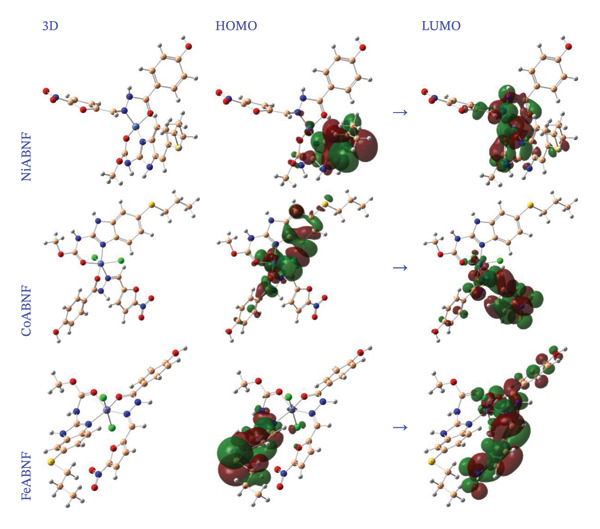
3D, HOMO, and LUMO of the FeABNF, CoABNF, and NiABNF complexes.

The study modeled the ligands by using DFT as illustrated in Figure (S.4) and considered their FeABNF, CoABNF, and NiABNF complexes in Figure 4. The FeABNF and CoABNF complexes were treated as octahedral geometry, while for the NiABNF complex, a tetrahedral geometry was assumed. The geometries of FeABNF, CoABNF, and NiABNF were confirmed by examining the bond angles listed in Table 4.

**TABLE 4:** Bond angles of the FeABNF, CoABNF, and NiABNF complexes.

FeABNF		CoABNF		NiABNF	
Angle	Value	Angle	Value	Angle	Value
Cl(64)–Fe(65)–Cl(63)	165.035	Cl(64)–Co(65)–Cl(63)	125.071	O(46)–Ni(63)–N(38)	100.373
Cl(64)–Fe(65)–O(46)	80.731	Cl(64)–Co(65)–O(46)	134.595	O(46)–Ni(63)–O(10)	116.255
Cl(64)–Fe(65)–N(38)	93.044	Cl(64)–Co(65)–N(38)	74.157	O(46)–Ni(63)–N(7)	116.830
Cl(64)–Fe(65)–O(10)	111.232	Cl(64)–Co(65)–O(10)	91.894	N(38)–Ni(63)–O(10)	114.829
Cl(64)–Fe(65)–N(7)	76.788	Cl(64)–Co(65)–N(7)	89.296	N(38)–Ni(63)–N(7)	118.840
Cl(63)–Fe(65)–O(46)	113.284	Cl(63)–Co(65)–O(46)	92.998	O(10)–Ni(63)–N(7)	90.859
Cl(63)–Fe(65)–N(38)	81.179	Cl(63)–Co(65)–N(38)	81.536	Ni(63)–O(46)–C(44)	124.221
Cl(63)–Fe(65)–O(10)	79.505	Cl(63)–Co(65)–O(10)	135.674	Ni(63)–N(38)–C(37)	122.992
Cl(63)–Fe(65)–N(7)	94.076	Cl(63)–Co(65)–N(7)	78.507	Ni(63)–N(38)–C(31)	127.748
O(46)–Fe(65)–N(38)	93.882	O(46)–Co(65)–N(38)	90.266	Ni(63)–O(10)–C(9)	110.117
O(46)–Fe(65)–O(10)	71.326	O(46)–Co(65)–O(10)	70.890	Ni(63)–N(7)–N(8)	109.075
O(46)–Fe(65)–N(7)	139.796	O(46)–Co(65)–N(7)	125.344	Ni(63)–N(7)–C(6)	126.068
N(38)–Fe(65)–O(10)	148.276	N(38)–Co(65)–O(10)	137.142		
N(38)–Fe(65)–N(7)	120.008	N(38)–Co(65)–N(7)	139.611		
O(10)–Fe(65)–N(7)	86.361	O(10)–Co(65)–N(7)	78.496		
Fe(65)–O(46)–C(44)	118.907	Co(65)–O(46)–C(44)	128.250		
Fe(65)–N(38)–C(37)	118.208	Co(65)–N(38)–C(37)	126.744		
Fe(65)–N(38)–C(31)	134.319	Co(65)–N(38)–C(31)	124.584		
Fe(65)–O(10)–C(9)	111.227	Co(65)–O(10)–C(9)	112.671		
Fe(65)–N(7)–N(8)	106.733	Co(65)–N(7)–N(8)	115.207		
Fe(65)–N(7)–C(6)	131.928	Co(65)–N(7)–C(6)	122.663		

Key frontier orbitals govern a molecule’s behavior: The lowest unoccupied molecular orbital and the highest occupied molecular orbital determine how a system reacts or resists change. When both HOMO and LUMO energies are negative, the molecule is considered stable. Table 5 lists the calculated HOMO and LUMO energies for the compounds studied. The HOMO–LUMO distributions are given together with the positive and negative regions of the ligands and their corresponding complexes in Figure (S.4) and Figure 4, respectively; red represents the negative phases, and green represents the positive phases.

**TABLE 5:** HOMO–LUMO derivative parameters (Δ*E* = energy gap, IP = ionization potential, EA = electron affinity, *χ* = electronegativity, CP = electronic chemical potential, *η* = chemical hardness, *ω* = electrophilicity index, *σ* = softness, Nu = nucleophilicity index) of the FeABNF, CoABNF, and NiABNF complexes.

Compounds	HOMO	LUMO	Δ*E*	IP	EA	*χ*	CP	*η*	*σ*	*ω*	Nu
NF	−6.57	−2.60	3.98	6.57	2.60	4.58	−4.58	1.99	0.25	5.28	0.19
AB	−5.52	−0.91	4.61	5.52	0.91	3.21	−3.21	2.30	0.22	2.24	0.45
FeABNF	−8.12	−5.70	2.41	8.12	5.70	6.91	−6.91	1.21	0.41	19.77	0.05
CoABNF	−4.58	−2.74	1.83	4.58	2.74	3.66	−3.66	0.92	0.55	7.31	0.14
NiABNF	−10.72	−9.34	1.38	10.72	9.34	10.03	−10.03	0.69	0.72	72.81	0.01

The HOMO level supplies information on the ability of a given species to donate electrons (Table 5). In this particular context, AB and NF can be referred to as moderate electron donors, with their values being −5.52 and −6.57 eV, respectively. If we analyze the entire set of complexes, the CoABNF model has an HOMO of −4.58 eV, indicating that it has the highest HOMO among all the complexes; thus, it has the highest nucleophilicity and therefore has the highest potential for interacting with electrophilic biological targets. On the contrary, the NiABNF complex, with a low HOMO of −10.72 eV, has the lowest HOMO.

Furthermore, regarding LUMO, it is defined by its ability to attract electrons. NF and AB at −2.60 and −0.91 eV, respectively, represent weaker electron acceptors than the complexes. Specifically, NiABNF displays the lowest LUMO level at −9.34 eV. Therefore, it is more electrophilic and is more likely to interact with biological nucleophiles. Meanwhile, FeABNF and CoABNF lie in between at −5.70 and −2.74 eV, respectively.

The energy gap ΔE, indicative of molecular reactivity, is smaller for more reactive compounds and, obviously, more potent biological compounds, as well. The ΔE values are smaller for more reactive compounds, with NiABNF displaying the smallest gap at 1.38 eV, followed by CoABNF at 1.83 eV, FeABNF at 2.41 eV, NF at 3.98 eV, and AB with the largest gap at 4.61 eV. These smaller gaps for the metal complexes suggest increased chemical reactivity, consistent with increased bioactivity predictions. The winner among these compounds, with the smallest energy gap and hence exhibiting maximum chemical reactivity, is NiABNF.

For instance, the ionization potential (IP) measures the required voltage to remove an electron from the compound. Thus, the lower the IP value, the higher the compound’s reactivity. Among the complexes, CoABNF has the lowest IP value at 4.58 eV; thus, it is ready to donate the electron, increasing the biological redox reactions. FeABNF and NiABNF have higher IP values of 8.12 and 10.72 eV, respectively; thus, they are stable and not susceptible to oxidation. Considering the ligands, AB has the lowest IP value at 5.52 eV compared to NF, which has an IP value of 6.57 eV. Electron affinity (EA) indicates the ease of accepting an electron by a particular species. NiABNF possesses the maximum EA, i.e., 9.34 eV, thus showing its maximum electrophilic character, which is likely to occur through electron acceptance. FeABNF possesses an EA of 5.70 eV, while CoAB NF has an EA of 2.74 eV. NF has an EA of 2.60 eV, while AB has an EA of 0.91 eV.

Electronegativity is a combination of IP and EA and provides a means of estimating the eagerness of a molecule to capture an electron. Of all the compounds, NiABNF possesses the highest electronegativity of 10.03 eV, which is followed by FeABNF with 6.91 eV, then NF with 4.58 eV, and finally CoABNF with 3.66 eV and AB with 3.21 eV. The high electronegativity of the NiABNF compound implies that the compound is eager to react with electron‐rich biological targets. The chemical potential (*μ*) of a compound represents the eagerness of the compound to give out an electron. NiABNF possesses the lowest potential of −10.03 eV among the compounds. It implies that the compound is very stable and is reluctant to give out an electron. FeABNF and NF are the next compounds, with electron‐giving potential of −6.91 and −4.58 eV, respectively. CoABNF and AB lie higher on the negative scale of *μ* with values −3.66 and −3.21 eV, respectively, implying that the compounds are more reactive.

Chemical hardness (*η*) stands for the extent to which the molecule holds onto its electrons. From the molecules listed, the hardest is AB, which records 2.30 eV. NF trails behind, recording 1.99 eV, followed by FeABNF, which records 1.21 eV, while CoABNF records 0.92 eV, followed by NiABNF, which records the lowest hardness of 0.69 eV, making it the softest molecule and therefore willing to share its electrons, which should enhance its bioactivity. Softness (*σ*) is a measure that acts in the opposite direction to hardness. In the case of softness, the highest softness is found to be 0.72 eV^−1^ by the NiABNF complex, followed by the CoABNF complex with a softness of 0.55 eV^−1^ and the FeABNF complex with a softness of 0.41 eV^−1^. NF is found to have a softness of 0.25 eV^−1^ followed by AB with a softness of 0.22 eV^−1^. In other words, essentially, the softer the complex, the better it will align itself to the environment, thus NiABNF being the softest or having the lowest *σ* of 0.72 eV^−1^, followed by CoABNF at 0.55 eV^−1^ and then FeABNF at 0.41 eV^−1^.

The electrophilicity index (*ω*) gives an approximation of the stabilization energy of a molecule after uptake of electrons. In this connection, the compound NiABNF shows an impressively high *ω* of 72.81, marking it out as a very strong electrophile indeed with a high biological reactivity. Other species, FeABNF and CoABNF, also exhibit very high *ω* values, namely, 19.77 and 7.31, respectively, while NF and AB are far behind at 5.28 and 2.24, respectively. In fact, *ω* identifies NiABNF beyond all doubt as the most biologically potent electrophile. Conversely, the nucleophilicity index (Nu) indicates the electron‐donating ability of a species. In this respect, the front‐runners are AB and NF with Nu values of 0.45 and 0.19, respectively, higher than the complexes. Among the latter, CoABNF is the most nucleophilic, Nu = 0.14, while for FeABNF and NiABNF, it is minimal, Nu = 0.05 and 0.01, respectively.

Better coordination translates to better electronic behavior and bigger biological potential. Thus, NiABNF has the smallest energy gap, highest electrophilicity, and highest softness and exhibits the best reactivity with the strongest biological effectiveness. CoABNF shows a good nucleophilic character and suitable softness in the second rank. FeABNF is reactive but less versatile from a biological point of view. The order for final biological activity is NiABNF > CoABNF > FeABNF > NF > AB.

Molecular electrostatic potential (MEP) diagram graphs are useful visualization tools that allow a clear understanding of how molecular bonds are distributed over a given surface. The graphs are convenient inasmuch as they aid in acquiring a clear understanding of how a given molecule might behave. It is also possible to get a clear understanding of how a given molecule could act in a particular biological capacity. The molecular structure graphs have a gradient of colors ranging from red to blue, which aid in the visualization of additional areas with varying potentials for attracting or repelling electrons. In this context, the areas highlighted in red are nuclei of high density, which usually include areas with oxygen and nitrogen. On the other hand, blue highlights usually represent the areas with a lack of electrons, which may include environments with hydrogen and metals, respectively.

By reviewing the optimized structures of the ligands and their respective complexes analyzed in the study, from the structures illustrated in Figure (S.5), patterns of charge separation are observed. Thus, the electropositive or electron‐rich regions around electronegative atom environments are highlighted, suggesting the potential sites for interaction with electrophilic amino acid environments that could be integral to target proteins. Conversely, blue environments near hydrogen atom environments have implications for hydrogen bonding donor environments that have the capacity to interact with the electronegative environments of carbonyl or carboxyl groups. Such electrostatic mapping, aside from providing further understanding of the reactivity of molecules, also enables the prediction of key pharmacophores.

### 3.3. Biological Activity

#### 3.3.1. Antibacterial Activity

**FIGURE 5:**
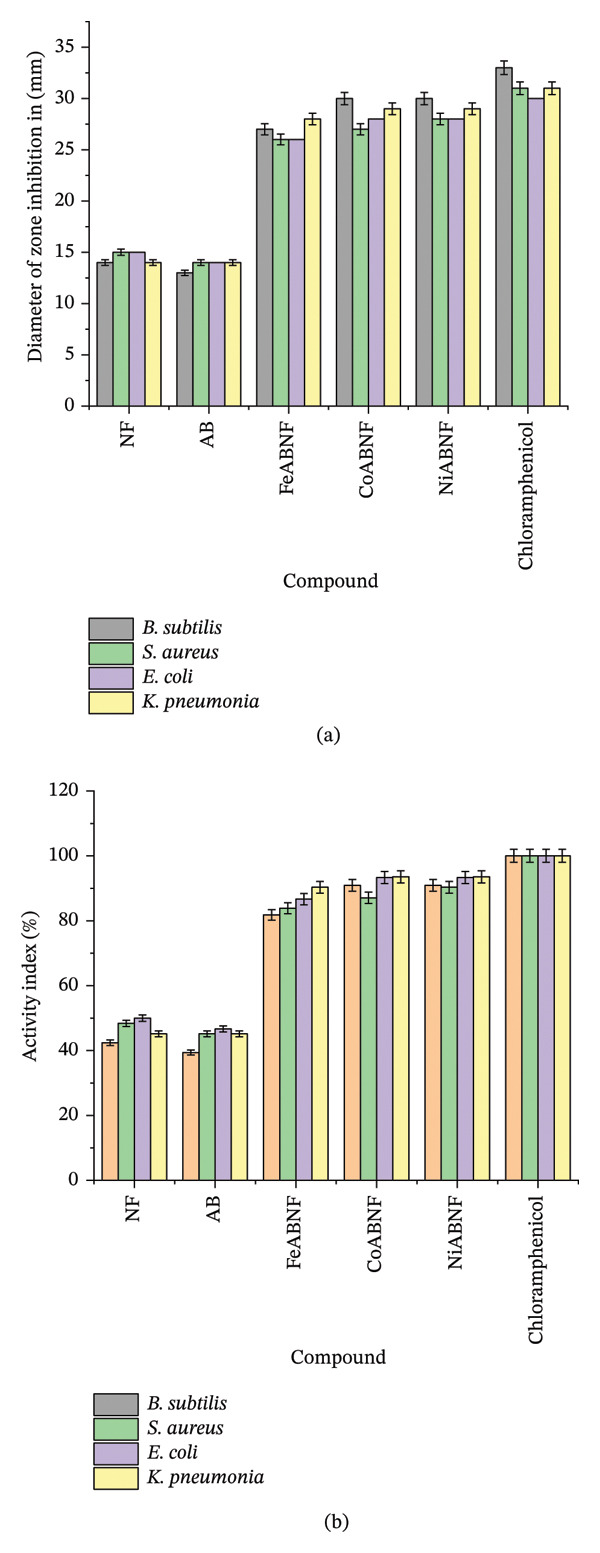
Antibacterial activity (a) zone of inhibition (mm), (b) activity index (%) of the FeABNF, CoABNF, and NiABNF complexes.

The antibacterial activity of ligands AB and NF, along with their metal complexes FeABNF, CoABNF, and NiABNF, was evaluated. Bacteria of both the Gram‐positive and Gram‐negative types were employed in the study. These bacteria comprise *B*. *subtilis* and *S*. *aureus* of the Gram‐positive variety, while *E*. *coli* and *K*. *pneumoniae* belong to the Gram‐negative category. Three parameters were used for the evaluation of activity, namely, the zone of inhibition, the AI in percent, and the data as exhibited in Tables (S.5) and (S.6). This is also represented in Figure 5.

The degree to which these areas of inhibition grow also corresponds to the antibacterial potential these compounds have to offer. Among the free ligands, it was observed that the compound NF and AB exhibited relatively low antibacterial properties; i.e., they inhibited the growth of the bacterial strains to the extent of only 14–15 mm. This effect was observed for both Gram‐positive and Gram‐negative bacterial strains. It was also seen that complexes of these free ligands had much higher antibacterial activity. Among these, FeABNF exhibited inhibition to the extent of 26–28 mm, the highest being for *K. pneumoniae* (28 mm). The two metal complexes, CoABNF and NiABNF, elongated the inhibition zones to the extent of 30 mm for the Gram‐positive bacterial strain *B. subtilis*, whereas for the Gram‐negative bacterial strains, the inhibition zone diameter was 28–29 mm.

The AI percent, too, confirms the inference that was made by the inhibition zones. NF and AB are placed in the moderate activity range, approximately 40%–50%, with a slight added potency against Gram‐negative bacteria. FeABNF shoots through, arriving at 82%–90%, demonstrating the effectiveness of coordination again. The CoABNF/NiABNF pair rules the roost, scoring approximately 91%–94%, breathing down the neck of chloramphenicol. The figures collectively suggest that CoABNF and NiABNF are very potent, acting through binding efficiency.

The pattern of antibacterial potency also remains the same, regardless of the dataset considered, and it shows that the potency of NiABNF and CoABNF is relatively similar while both surpass the potency of FeABNF, NF, and AB, in which the last two compounds show equal potency. There is, however, a significant increase in potency after the formation of complexes. It is of particular note that the potency of NiABNF and CoABNF is very similar, indicating that both compounds can be considered potent antibacterial agents.

The results obtained in DFT analysis offer a clear explanation for the biological activity exhibited by the compounds. It appears that among all the tested compounds, NiABNF has the lowest Δ*E* = 1.38 eV, the highest electrophilicity *ω* = 72.81, and the highest softness *σ* = 0.72. This positive combination results in a high reactivity and top‐notch ability to accept electrons. It is also noteworthy that CoABNF has also low Δ*E* = 1.83 eV and high softness *σ* = 0.55, as well as relatively high electrophilicity *ω* = 7.31. This results in a positive reactivity. On the contrary, it has been observed that in the case of FeABNF, Δ*E* = 2.41, which is higher than that of other tested compounds, along with lower softness. It is therefore not surprising that this compound is less active. It is also evident that in the cases of NF and AB, Δ*E* is high, while electrophilicity is relatively weaker, resulting in low biological activity.

#### 3.3.2. Antifungal Activity

**FIGURE 6:**
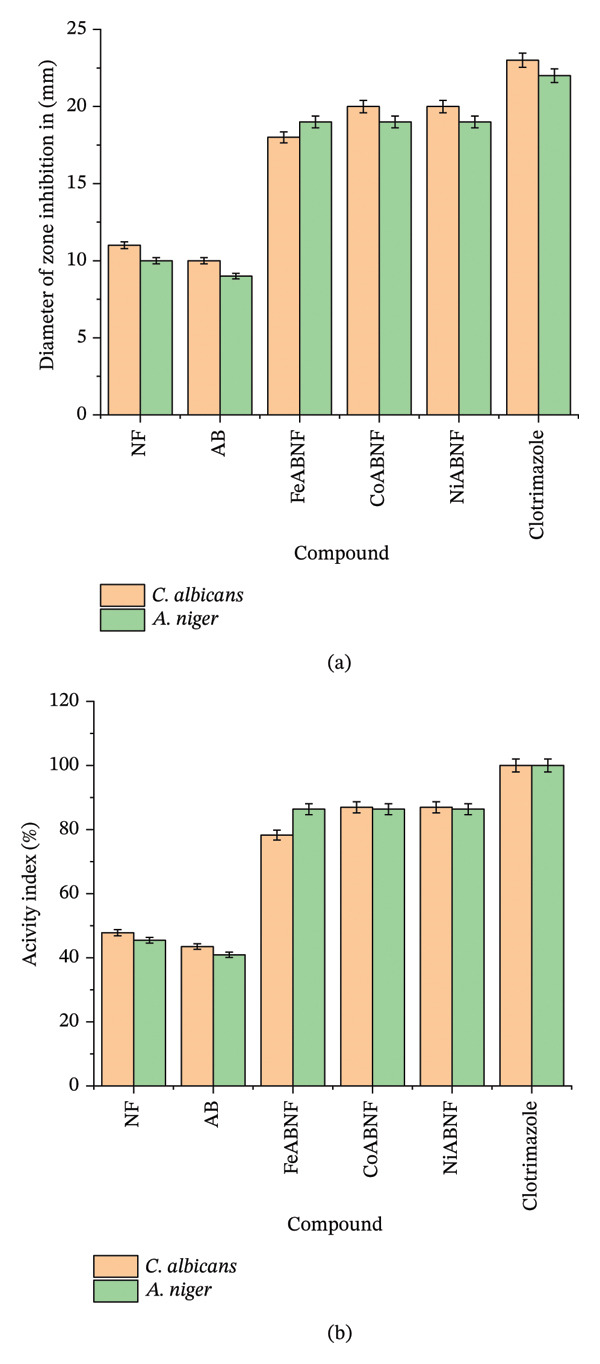
Antifungal activity. (a) zone of inhibition (mm), (b) activity index (%) of the FeABNF, CoABNF, and NiABNF complexes.

Antifungal potential of the ligands AB and NF, and their metal complexes FeABNF, CoABNF, and NiABNF was evaluated against the antifungal species *C*. *albicans* and *A*. *niger*. Three parameters were used to evaluate the antifungal potency of the compounds: zone of inhibition (mm), as shown in Table (S.7); AI, as shown in Table (S.8); and the data are represented graphically in Figure 6.

The antifungal test, which is in accordance with inhibition zone, demonstrates that the free ligands NF and AB have poor activity. The inhibition zone size for these compounds is approximately 10–11 and 9–10 mm, respectively. However, when these ligands are in their complexes, their activity is increased. The activity of FeABNF is moderately high at approximately 18–19 mm. The results obtained for CoABNF and NiABNF are significant. This is because these compounds have the largest inhibition zone, approximately 20 mm for both species of fungi, i.e., *C*. *albicans* and *A. niger*. This is almost similar to the activity possessed by clotrimazole, which is in the range of 23–22 mm.

The AI shows the performance of each compound against the standard antifungal drug clotrimazole. NF shows a moderate effect at 46%–48%. AB shows practically zero activity at all, 0%. FeABNF makes a clear jump to about 78%–86% activity, while the metal complexes CoABNF and NiABNF hit the top with about 87%, effective against both fungi. The reasonable increase in the AI may indicate that the formation of metal complexes increases the bioavailability and potency of the ligand.

The antifungal tests have also yielded a similar result. The NiABNF and CoABNF complexes have almost equal efficacy and are more potent than the FeABNF complex. The FeABNF complex is more potent than the NF and the free ligands. The nickel and the cobalt complexes are almost equally potent but closer to the standard drug. The free ligands have little or almost no efficacy. The table succinctly indicates the superior effect of the metal complexes.

The antifungal behavior follows a pattern with the given DFT descriptors. NiABNF stands out with a smaller Δ*E* of only 1.38 eV, a higher electrophilicity index *ω* of 72.81, and a higher softness *σ* of 0.72, making this compound more reactive. Therefore, this compound is more likely to interact with biological systems. CoABNF also exhibited excellent antifungal properties with a small Δ*E* of 1.83 eV, a satisfactory level of softness (*σ* = 0.55), and a moderate electrophilicity index (*ω* = 7.31). On the other hand, FeABNF also exhibited excellent antifungal properties although to a lesser degree, given its higher Δ*E* of 2.41 eV and a lower electrophilicity index of 19.77, confirming its lower reactivity. NF and AB have a wide Δ*E* gap with a very low *ω*, making these two compounds less reactive, resulting in a low antifungal effect.

The key according to the chelation theory for more effective antifungal properties lies in chelate formations. By forming more stable chelates, the compound reduces its polarity since it tends to share some of its positive charges with other donor atoms, thereby making it more lipophilic. Veing more lipophilic, this allows the compound to more readily pass through fungal membranes. In this context, CoABNF and NiABNF are more effective in that they form more lipophilic complexes compared to FeABNF, which transforms into chelates that are more effective biological agents. Moreover, chelation is also known to enhance the capacity for which these antifungal solutions can interact with fungal targets.

#### 3.3.3. Anti‐inflammatory

**FIGURE 7:**
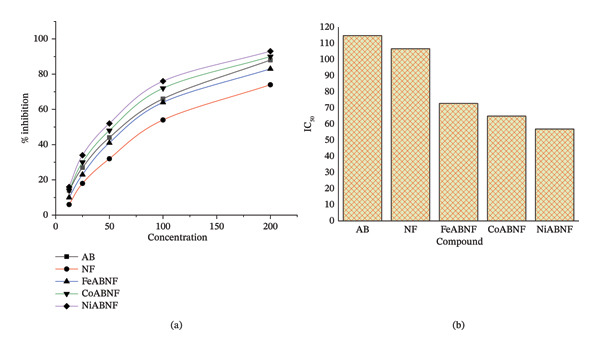
Anti‐inflammatory of the FeABNF, CoABNF, and NiABNF (a) inhibition percentage (b) IC50.

The anti‐inflammatory effect obviously increases when metal binding occurs, as reflected in the percentage inhibition across Table (S.9) and Figure 7. Free ligands AB and NF are less effective at all tested concentrations, culminating in a maximum of 88% and 74% inhibition, respectively, at 200 μM. In contrast, the metal complexes FeABNF, CoABNF, and NiABNF attain inhibitions of 83%, 90%, and the highest with 93%. At all dosage levels, NiABNF is leading, followed by CoABNF and FeABNF. This trend indicates that the coordination with metals significantly enhances the anti‐inflammatory ability of the ligands, with NiABNF being the most potent among all dosages tested.

These IC_50_ values correlate with what the percentages based on inhibitor concentration were already making us understand. NiABNF has the lowest IC_50_ at 56.97 μM, which indicates potency, followed by CoABNF at 64.98 μM. After this comes FeABNF at 72.87 μM. AB and NF are far back at 114.86 μM and 106.74 μM, respectively. This illustrates that indeed, there existed an inverse relationship between IC_50_ and efficacy; that is, the lower the IC_50_, the higher efficacy. So, NiABNF has the highest potency, followed by a significant potency increase for CoABNF and FeABNF.

The order in which anti‐inflammatory activity is expressed follows a pattern: NiABNF > CoABNF > FeABNF > NF > AB. This is true for the degree of inhibition as well as the IC50 values. However, in all the compounds under consideration, the anti‐inflammatory activity is increased to a significant degree upon coordination with the transition metals. Among the compounds under consideration for anti‐inflammatory activity, the most promising is the NiABNF.

The anti‐inflammatory efficacy follows a similar pattern with respect to these reactivity descriptors provided by DFT. Among all, NiABNF was distinguished by its lowest Δ*E* value (1.38 eV), highest electrophilicity index (*ω* = 72.81), and highest *σ* value (0.72). A clear picture of a highly reactive and versatile molecular structure ready to efficiently act on biological targets is formed. CoABNF, with a moderate Δ*E* value (1.83 eV) and a proper balance between *ω* (7.31) and *σ* (0.55) values, also indicates excellent efficacy. FeABNF is also effective, although its higher Δ*E* (2.41 eV) and lower *σ* (0.41) values suggest that, unlike others, its activity might be limited with respect to biological targets. By comparison, ligands AB and NF are much less likely to initiate anti‐inflammatory responses, given their worse electronic properties.

### 3.4. Molecular Docking

In the first instance, we conducted a validation run that brought together redocking and the superimposing of native ligands in order to evaluate whether the docking method used was valid. The two cocrystallized ligands were initially bound to 4DUH and 6LU7. These were 4‐([4′‐methyl‐2′‐(propanoylamino)‐4,5′‐bi‐1,3‐thiazol‐2‐yl]amino)benzoic acid and n‐[(5‐methylisoxazol‐3‐yl)carbonyl]alanyl‐l‐valyl‐n1‐(4‐(benzyloxy)‐4‐oxo‐1‐([2‐oxopyrrolidin‐3‐yl]methyl)but‐2‐enyl)‐l‐leucinamide. The process worked quite accurately to produce a very accurate replica of the binding positions of the natural ligands with RMSD of 1.074 and 1.112, respectively. The binding position matched perfectly with that of the native ligands (Figure (S.6)). These results support the use of the docking approach in upcoming investigations of related drugs by validating its capacity to accurately predict binding modes.

#### 3.4.1. Molecular Docking Against 4DUH

**FIGURE 8:**
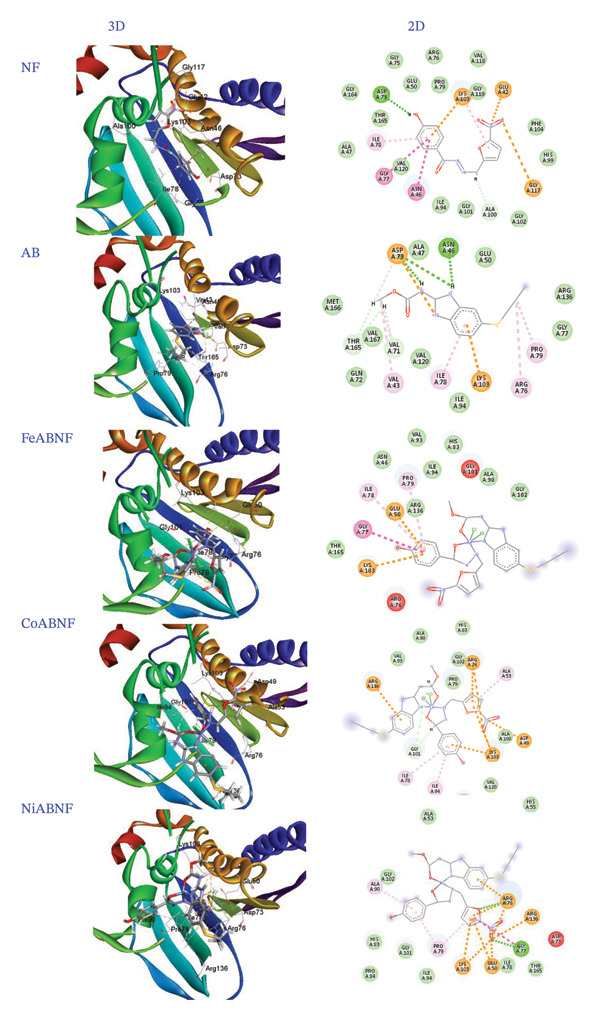
3D molecular docking pose of the AB, NF, and their FeABNF, CoABNF, and NiABNF complexes against 4DUH receptor.

To assess how the chemicals under investigation interacted with the target protein, DNA gyrase B (PDB ID: 4DUH), molecular docking simulations were conducted (Table (S.10), and Figure 8).

NF reveals a modest binding to DNA gyrase B with a binding energy of about −7.10 kcal/mol. It perfectly couples to the active site, forming significant hydrogen bonds to ASP73 at 2.20 Å and to ALA100 at 2.17 Å, which enhances its specificity. Electrostatic tugs toward GLY117 (4.93 Å), GLU42 (3.91 Å), and LYS103 (4.94 Å) help lock the ligand in place, while hydrophobic contacts to ILE78 (4.44 Å) and LYS103 (3.91 Å) enhance its nonpolar compatibility. On the other hand, AB binds DNA gyrase B more loosely at around −6.90 kcal/mol. It forms hydrogen bonds with GLU50 (2.77 Å) and GLY77 (3.00 Å), besides several hydrophobic interactions by PRO79, VAL43, VAL120, VAL167, and ILE78 (around 4.5–4.8 Å). There are also electrostatic interactions with GLU50 and LYS103, though less intricate.

This binding energy is drastically increased by FeABNF to about −8.10 kcal/mol, somewhat binding to LYS10 at 4.23 Å and GLU50 at 4.03 Å through electrostatic interactions. The binding pocket complementarity is also aided by hydrophobic interactions with ILE78 (5.37 Å) and PRO79 (4.87 Å). The Fe coordination center would provide considerable structural rigidity and a better electronic complement. CoABNF also has a strong binding energy of −8.60 kcal/mol. This is through the presence of an unmistakable binding interaction profile characterized by hydrogen bond interactions with GLY101 and ARG76, which help in strong binding through the presence of appreciable bond lengths of 2.16 and 3.46 Å, respectively. The electrostatic interactions with ARG76, LYS103, ASP49, and ARG136 assist the ligand in appreciable recognition. A solid hydrophobic cluster of ALA53, ILE78, ILE94, and LYS103 through multiple interactions aids in the ligand conformational stability. NiABNF shows the strongest affinity, with an affinity energy of −8.90 kcal/mol due to the strong H‐bonding between ARG76 (3.02 Å) and GLY77 (2.63 Å), thus making it easier to hit the active site more specifically. Other electrostatic interactions with ARG76, ARG136, LYS103, and GLU50, with distances varying between 3.79 and 5.45 Å, indicate more polar residue contributions to the stabilizing ability of this compound. The hydrophobic effects of PRO79, ILE78, and ALA90, with distances not exceeding 5.47 Å, will only serve to enhance affinity through associated van der Waals forces.

The high DNA‐binding affinity of the NiABNF complex, as revealed by molecular docking studies against the 4DUH DNA structure, can be attributed to the combined effect of strong π–π stacking interactions between the aromatic moieties of the complex and DNA base pairs, along with favorable electrostatic interactions with the phosphate backbone. Furthermore, coordination to the Ni(II) center enhances the planarity and rigidity of the ligand framework, facilitating efficient intercalation into the DNA helix. Additional stabilization arises from hydrogen bonding and van der Waals interactions, resulting in a more negative binding energy and stronger binding affinity compared to the free ligand and other studied complexes.

The binding sequences remain constant in all cases: NiABNF > CoABNF > FeABNF > NF > AB. This is true for all possible energies and all patterns of interaction. Several interactions, both polar and hydrophobic, are observed in both NiABNF and CoABNF, which influence both enthalpic and entropic contributions, while AB and NF make fewer high‐stability contacts with higher‐energy binding, providing limited biological opportunity. The stronger performance of these docking complexes could result from improvements provided by metals in terms of geometry, charge, and ligand flexibility.

These docking outcomes correlate convincingly with those of the *in vitro* antibacterial tests (Tables (S.5) and (S.6)). Among these compounds, NiABNF and CoABNF recorded the lowest binding energy values and highest interaction networks; in other words, they had the largest inhibitory zone and highest indices of activity, close to 91%–94%. Although FeABNF scored low in interactions, its bioactivity is moderate. NF and AB compounds, however, recorded poor antibacterial properties due to their low interaction. Therefore, these docking outcomes satisfactorily predict *in vitro* efficacy and highlight enhanced bioactivity upon metal complexation.

DFT analysis has been performed to support the docking study together with the observed biological trends. NiABNF possesses the smallest Δ*E*, 1.38 eV, and by far the highest electrophilicity, *ω* = 72.81, and the largest softness, *σ* = 0.72, indicating ready adaptation to the target and easy electron exchange therein. The next is CoABNF, with a low Δ*E* of 1.83 eV and good softness, *σ* = 0.55. Such features account for good electrostatic and hydrogen bond interactions during docking. FeABNF assumes a middle position with respect to reactivity, Δ*E* = 2.41 eV, that fits its overall moderate docking performance and bioactivity. By contrast, NF and AB possess higher Δ*E* and lower *ω*, making them electronically less suitable for efficient binding—a fact that agrees with their overall weaker docking scores and biological data.

#### 3.4.2. Molecular Docking Against 6LU7

**FIGURE 9:**
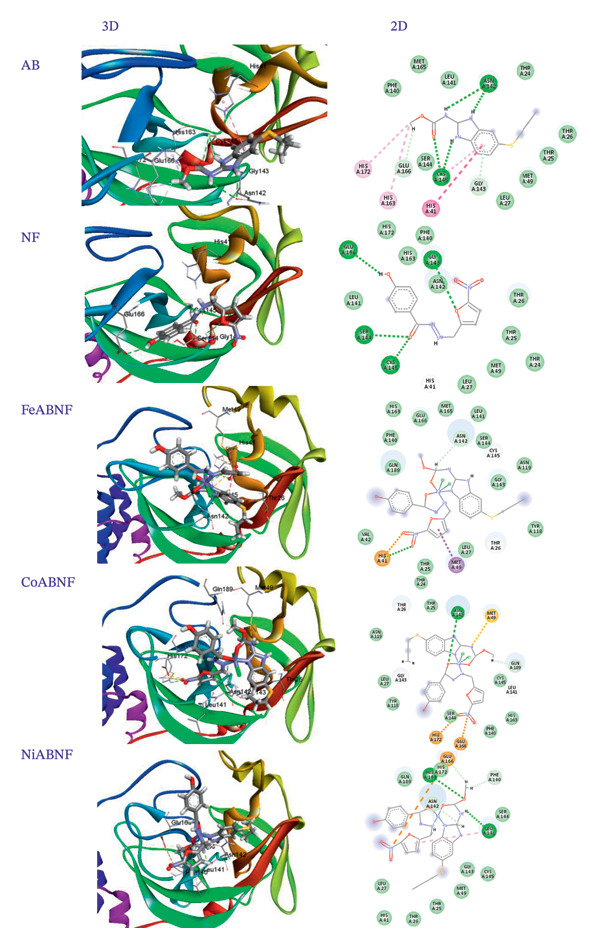
Three‐dimensional molecular docking pose of the AB, NF, and their FeABNF, CoABNF, and NiABNF complexes against the 6LU7 receptor.

To assess how the chemicals under investigation interacted with the target protein, PDB ID: 6LU7, molecular docking simulations were conducted (Table (S.11), and Figure 9).

The ligand, AB, had moderate affinity for the 6LU7 receptor, with a binding energy of −6.40 kcal/mol. It made four hydrogen bonds with significant active‐site residues CYS145, ASN142, GLU166, and GLY143, with bond lengths of 3.48, 2.45, 2.81, and 2.88 Å, respectively. It formed hydrophobic interactions with important residues HIS41, HIS163, HIS172, and CYS145, with bond lengths between 4.62 and 5.92 Å. On the other hand, NF demonstrated strong affinity compared to AB, with a binding energy of −6.80 kcal/mol. It formed robust hydrogen bonds with significant residues, including GLY143, SER144, CYS145, and GLU166, with bond lengths of 2.62, 2.58, 2.10, and 2.53 Å.

The FeABNF complex displayed the highest binding activity with an affinity of −8.70 kcal/mol, supported by the interactions it formed with the receptor. It had an electrostatic contact with HIS41 at a distance of 5.07 Å and formed two hydrogen bonds with the same residue at a distance of 2.93 and 2.90 Å, respectively. It also formed a hydrogen bond with ASN142 at 2.21 Å, and a hydrophobic contact was formed with MET49 at 3.01 Å. The binding affinity of the CoABNF complex was the highest at −9.10 kcal/mol, supported by the strong interaction it formed with the receptor. It had an electrostatic contact with both HIS172 at a distance of 5.37 Å and GLU166 at a distance of 2.93 Å, and it formed hydrogen bonds with ASN142 at 2.92 Å and HIS172 at 2.46 Å. Among the different compounds tested for activity, the highest activity was shown by the NiABNF compound, whose binding activity was the highest at −9.40 kcal/mol, indicating strong potential for inhibition. It had formed hydrogen bonds with various amino acid residues such as GLU166 at 1.99 Å and 5.15 Å, HIS163 at 2.35 Å, LEU141 at 1.87 and 4.74 Å, ASN142 at 2.70 Å, and PHE140 at 1.54 Å, thus spanning a considerable part of the target site.

In summary, it is seen that the binding affinity of the metal complexes is much greater compared to free ligands due to a greater ability to coordinate, resulting in stronger molecular interactions in the active site. The binding energies of these complexes have been given in a descending order of their values: NiABNF (−9.40), CoABNF (−9.10), FeABNF (−8.70), NF (−6.80), and AB (−6.40). It is also important to note that all these complexes have strong binding affinities with key active‐site residues of the Mpro enzyme, namely, HIS41, CYS145, GLU166, and ASN142, making these complexes effective in designing a drug targeting respiratory viruses like COVID‐19. Notably, in comparison with free ligands, the complexes have a much better binding affinity, with NiABNF being the best, suggesting that these complexes underscore their potential as respiratory enzyme inhibitors.

## 4. Conclusion

This study describes the successful synthesis and full characterization of three new complexes of transition metal elements identified as FeABNF, CoABNF, and NiABNF derived from the AB and NF ligands. Using a detailed set of physicochemical analyses and investigations, it was established that the new complexes possessed substantial structural integrity. Their spectral and magnetic properties suggested their coordination spheres were nonidentical: octahedral for Fe(III) and Co(II) and tetrahedral for Ni(II). They demonstrated good thermal stability above 300°C and near‐neutral pH of 6–8. DFT also provided significant electronic structure insights. From the three candidates, NiABNF excelled in terms of its reactivity caused by a narrow HOMO–LUMO gap of 1.38 eV and high electrophilicity characterized by *ω* = 72.81, as well as significant structural flexibility indicated by *σ* = 0.72 eV^−1^. This correlated with its strong biological activity. The antimicrobial results demonstrate that coordination of the ligand to metal ions improves antimicrobial activity in most cases, although the effect is not uniform across all tested micro‐organisms. These findings highlight the importance of metal ion selection in tuning biological activity. Specifically, NiABNF and CoABNF were more active against various micro‐organisms, including Gram‐positive and Gram‐negative bacteria, as well as the fungi *C. albicans*, *A. niger*, compared to the free ligands. For anti‐inflammatory screening, the potency of NiABNF was again noteworthy, reaching 93% and an IC_50_ of 56.97 μM, thus demonstrating the potential of chelation to enhance potency. Molecular docking provided insight into how this was achieved, with NiABNF showing the strongest affinity toward DNA gyrase B with a ΔG of −8.90 kcal/mol, driven by hydrogen bonding as well as hydrophobic effects. It was demonstrated that there are significant improvements in the potential binding ability of the metal complexes, especially NiABNF, as compared to their free ligands, indicating their potential application for tackling respiratory viral diseases, such as COVID‐19, among others. Therefore, DFT was shown to be valid, as assembled through experimental data.

**Nomenclature**
AB: Albendazole
NF: Nifuroxazide
DFT: Density functional theory
HOMO: Highest occupied molecular orbital
LUMO: Lowest unoccupied molecular orbital
MIC: Minimum inhibitory concentration
CDK: Cyclin‐dependent kinase
IC_50_
: Half maximal inhibitory concentration
UV–vis: Ultraviolet–visible spectroscopy
IR: Infrared spectroscopy
NMR: Nuclear magnetic resonance
DNA: Deoxyribonucleic acid
ROS: Reactive oxygen species
DMSO: Dimethyl sulfoxide
Mpro: Main protease
AI%: Activity index

## Author Contributions

Yousef Aldabayan S.: investigation, resources, formal analysis, funding acquisition, writing–original draft, and writing–review and editing. Hany M. Abd El‐Lateef: investigation, resources, formal analysis, funding acquisition, writing–original draft, and writing–review and editing. Mai M. Khalaf: investigation, resources, formal analysis, funding acquisition, writing–original draft, and writing–review and editing. Aly Abdou: investigation, methodology, resources, formal analysis, data curation, funding acquisition, writing–original draft, and writing–review and editing.

## Funding

This work was supported by the Deanship of Scientific Research, Vice Presidency for Graduate Studies and Scientific Research, King Faisal University, Saudi Arabia (Grant No. KFU262132).

## Conflicts of Interest

The authors declare no conflicts of interest.

## Supporting Information

Additional supporting information can be found online in the Supporting Information section.

## Supporting information


**Supporting Information** List of supplementary figures: Figure (S.1): FT–IR spectra of the FeABNF, NiABNF, and CoABNF complexes. Figure (S.2): The stoichiometry of the NF, AB, FeABNF, NiABNF, and CoABNF. Figure (S.3): pH stability curve of the FeABNF, NiABNF, and CoABNF complexes. Figure (S.4): The 3D, HOMO, and LUMO of the nifuroxazide (NF) and albendazole (AB ligands. Figure (S.5): The MEP of the nifuroxazide (NF) and albendazole (AB) ligands FeABNF, CoABNF, and NiABNF. Figure (S.6): Three‐dimensional representation of the superimposition of the cocrystallized (green) and the redocked (red) ligand of (a): PDB ID: 4DUH, and (b): PDB ID 6LU7. List of supplementary tables: Table (S.1): Physical properties of the FeABNF, NiABNF, and CoABNF complexes. Table (S.2): conductivity of the FeABNF, NiABNF, and CoABNF complexes. Table (S.3): UV–vis spectra of the FeABNF, NiABNF, and CoABNF complexes. Table (S.4): Effective magnetic moment of the FeABNF, NiABNF, and CoABNF complexes. Table (S.5): Antibacterial activity as the diameter of zone inhibition in mm. Table (S.6): Antibacterial activity as the activity index (%). Table (S.7): Antifungal activity as the diameter of zone inhibition in mm. Table (S.8): Antifungal activity as the activity index (%). Table (S.9): Anti‐inflammatory results as mean percentage inhibition and IC_50_ of the compounds studied. Table (S.10): Molecular docking data of the AB, NF, and their FeABNF, CoABNF, and NiABNF complexes against the 4DUH receptor. Table (S.11): Molecular docking data of the AB, NF, and their FeABNF, CoABNF, and NiABNF complexes against the 6LU7 receptor.

## Data Availability

The data that support the findings of this study are available in the supporting information of this article.
