# Supplementary material for: Synthesis, Structural Characterization, and DFT Studies of Fe(III), Co(II), and Ni(II) Mixed‐Ligand Complexes With Albendazole and Nifuroxazide With Molecular Docking Against Respiratory Pathogen Targets
Source: Bioinorg Chem Appl. 2026 Jun 19;2026:8879576. doi: 10.1155/bca/8879576 (PMC13282283; doi:10.1155/bca/8879576)
Supplement: Supplementary file 1 — Supporting Information List of supplementary figures: Figure (S.1): FT–IR spectra of the FeABNF, NiABNF, and CoABNF complexes. Figure (S.2): The stoichiometry of the NF, AB, FeABNF, NiABNF, and CoABNF. Figure (S.3): pH stability curve of the FeABNF, NiABNF, and CoABNF complexes. Figure (S.4): The 3D, HOMO, and LUMO of the nifuroxazide (NF) and albendazole (AB ligands. Figure (S.5): The MEP of the nifuroxazide (NF) and albendazole (AB) ligands FeABNF, CoABNF, and NiABNF. Figure (S.6): Three‐dimensional representation of the superimposition of the cocrystallized (green) and the redocked (red) ligand of (a): PDB ID: 4DUH, and (b): PDB ID 6LU7. List of supplementary tables: Table (S.1): Physical properties of the FeABNF, NiABNF, and CoABNF complexes. Table (S.2): conductivity of the FeABNF, NiABNF, and CoABNF complexes. Table (S.3): UV–vis spectra of the FeABNF, NiABNF, and CoABNF complexes. Table (S.4): Effective magnetic moment of the FeABNF, NiABNF, and CoABNF complexes. Table (S.5): Antibacterial activity as the diameter of zone inhibition in mm. Table (S.6): Antibacterial activity as the activity index (%). Table (S.7): Antifungal activity as the diameter of zone inhibition in mm. Table (S.8): Antifungal activity as the activity index (%). Table (S.9): Anti‐inflammatory results as mean percentage inhibition and IC50 of the compounds studied. Table (S.10): Molecular docking data of the AB, NF, and their FeABNF, CoABNF, and NiABNF complexes against the 4DUH receptor. Table (S.11): Molecular docking data of the AB, NF, and their FeABNF, CoABNF, and NiABNF complexes against the 6LU7 receptor. [file BCA-2026-8879576-s001.doc]

**Supplementary data**

**Synthesis, Structural Characterization and DFT Studies of Fe(III), Co(II), and Ni(II) Mixed-Ligand Complexes with Albenadazole and Nifuroxazide with Molecular Docking Against Respiratory Pathogen Targets**

Yousef S Aldabayan^1*^, Hany M. Abd El-Lateef^2*^, Mai M. Khalaf^2^, Aly Abdou^3*^

^1^Department of Respiratory Care, King Faisal University, AL Ahsa 31982, Saudi Arabia

^2^Department of Chemistry, College of Science, King Faisal University, Al-Ahsa 31982, Saudi Arabia

^3^Department of Chemistry, Faculty of Science, Sohag University, Sohag 82524, Egypt

* Corresponding authors:

[yaldabayan@kfu.edu.sa](mailto:yaldabayan@kfu.edu.sa) (Y. S. Aldabayan) ; [hmahmed@kfu.edu.sa](mailto:hmahmed@kfu.edu.sa) (H. M. Abd El-Lateef.); [aly_abdou@science.sohag.edu.eg](mailto:aly_abdou@science.sohag.edu.eg) (A. Abdou)

**Characterization Techniques**

Elemental analysis for C, H, and N was performed using a Perkin-Elmer 2408 elemental analyzer. Metal analysis (%M) was performed using Atomic Absorption Spectroscopy (AAS) with a Perkin-Elmer 2380 Atomic Absorption Spectrophotometer. IR spectral studies were performed using a BRUKER FT-IR model 8101 spectrophotometer to study the influence of coordination on the functional groups present in the ligands and their metal complexes. Electronic spectral studies were performed using a Jasco V-750 UV-Vis spectrophotometer. Mass spectral studies were performed using Thermo Scientific GCMS model ISQ spectrophotometer. Thermoanalytical studies were performed using a Shimadzu Analyzer model 60 H at a heating rate of 10 °C per minute. Molar conductivity measurements were performed using a JENWAY conductivity meter model 4320 on 10^-3^ M solutions in ethanol. Magnetic susceptibility measurements were performed using the powder magnetic susceptibility method with a Bartington susceptibility instrument model 4320. Finally, the stoichiometry of the metal complexes was determined by the spectrophotometric method of continuous variation.

**Stoichiometry of the synthesized mixed-ligand complexes**

The continuous variation method was used to establish the composition of the ternary M: NF: AB) complexes [1, 2]. The molar fractions of the two components varied continuously, keeping their total concentration constant in the presence of a significant excess of the third component. Under these conditions, the ternary system was modified to a pseudo-binary system [1, 2].

Firstly, the stoichiometry of M: NF in the presence of SA as mixed ligands must be determined. A series of solutions containing different ratios of M: NF were prepared (in the presence of excess AB), keeping the total concentration of both M ion and NF constant. The ratio of M: NF, was determined from the relationship between absorbance (Abs) (at the λ_max_ of the target complex) and mole fraction of NF (NF /(NF +M)).

Secondly, the ratio of M: AB was determined as described above in the presence of excess (NF). A series of solutions containing different ratios of M: AB, were prepared (in the presence of an excess of NF), keeping the total concentration of both M ion and AB constant. The ratio of M: AB, was determined from the relationship between absorbance (Abs) (at the λ_max_ of the target complex) and mole fraction of CP: (AB /(AB +M)).

The results proved the 1:1:1 (M: NF: AB.) ternary complexes were formed for FeABNF, CoABNF, and NiABNF complexes.

**Biological assay**

**Antibacterial Activity**

The in vitro antibacterial activity of the synthesized compounds was evaluated using the disc diffusion method against four pathogenic bacterial strains, two Gram-positive: *B. subtilis* and *S. aureus*, and two Gram-negative: *E. coli* and *K. pneumoniae*.

Each bacterial strain was cultured to a concentration of approximately 0.5 × 10⁶ CFU/mL, and a 100 µL aliquot was uniformly swabbed onto Mueller Hinton Agar (MHA) plates. The test compounds were prepared at a concentration of 50 mg/mL in dimethyl sulfoxide (DMSO). Sterile 6 mm paper discs were impregnated with 20 µL of each test solution and gently placed onto the surface of the inoculated plates. The plates were then inverted and incubated at 37 °C for 24 hours under aerobic conditions. Following incubation, the zones of inhibition around each disc were measured in millimeters (mm), reflecting the antibacterial efficacy of the compounds. DMSO was used as a negative control, confirming no inherent antimicrobial activity. The standard antibiotic chloramphenicol was employed as a positive control for comparative analysis.

**Antifungal Activity**

The antifungal potential of the studied compounds was assessed against two fungal pathogens: *C. albicans* and *A. niger*, using the disc diffusion assay. Sabouraud Dextrose Agar (SDA) plates were inoculated with standardized fungal spore suspensions (1 × 10⁵ CFU/mL).

Sterile discs (6 mm) were saturated with 20 µL of each compound solution (50 mg/mL in DMSO) and positioned on the agar surface. The inoculated plates were incubated under suitable conditions: 37 °C for 28 hours. After incubation, the diameter of the inhibition zones formed around each disc was measured to evaluate antifungal activity. Clotrimazole served as the standard antifungal drug, and DMSO was used as a negative control.

**Activity Index (%)**

The antimicrobial efficacy of each test compound was further quantified by calculating the Activity Index (AI%), comparing the performance of each compound to its corresponding standard drug (chloramphenicol for bacteria and clotrimazole for fungi). The AI% was determined using the formula: Activity Index (AI%)= [Zone of Inhibition of Test Compound (mm) / Zone of Inhibition of Standard (mm)]×100.

**Anti-inflammatory Activity**

The anti-inflammatory potential of the synthesized compounds was assessed in triplicate using the egg albumin denaturation assay, with ibuprofen employed as the standard reference drug. Stock solutions of the test compounds were prepared at concentrations of 12.5, 25, 50, 100, and 200 µg/mL in DMSO using phosphate buffer (0.2 M, pH 6.4) as diluent. To each 4 mL of the test solution, 1 mL of freshly prepared egg albumin (diluted 1:9 with distilled water) was added to yield the final assay mixture. The mixtures were incubated at 37 °C for 15 minutes, followed by heat-induced denaturation in a water bath at 70 °C for 5 minutes. After cooling to ambient temperature, turbidity due to denatured protein was observed in the reaction mixtures.

The absorbance of each solution was recorded at 660 nm using a UV-Visible spectrophotometer. The extent of inhibition of protein denaturation was determined relative to a control sample (containing egg albumin and buffer but no test compound) using the following equation: Percentage Inhibition=[1-(X/Y)]×100. Where: X = Absorbance of the sample containing the test compound, Y = Absorbance of the control sample. The percentage inhibition values were used to generate dose-response curves, from which IC_50_ values (the concentration required to inhibit 50% of albumin denaturation) were calculated to quantify the anti-inflammatory efficacy of the compounds using <https://www.aatbio.com/tools/ic50-calculator>.

|  |
| --- |
| Fig. (S.1): FT-IR spectra of the FeABNF, NiABNF, and CoABNF complexes |

|  |  |
| --- | --- |
| Fig. (S.2): The Stoichiometry of the NF, AB, FeABNF, NiABNF, and CoABNF | |

|  |
| --- |
| Fig. (S.3): pH stability curve of the FeABNF, NiABNF, and CoABNF complexes. The stability of the complexes was monitored over a pH range of 2.0 to 10.0 in an aqueous universal buffer system. |

|  | 3D | HOMO |  | LUMO |
| --- | --- | --- | --- | --- |
| NF | 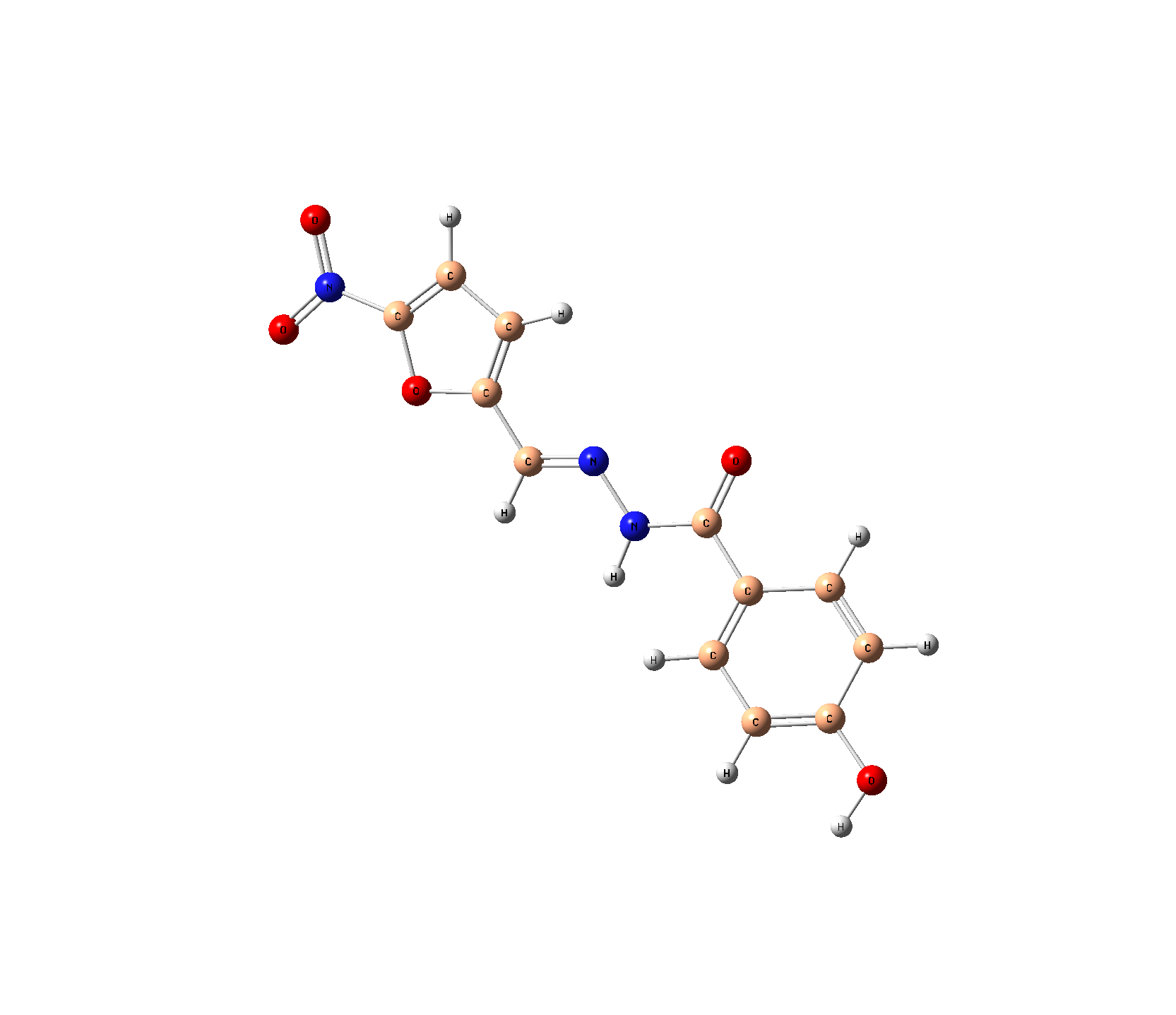 | 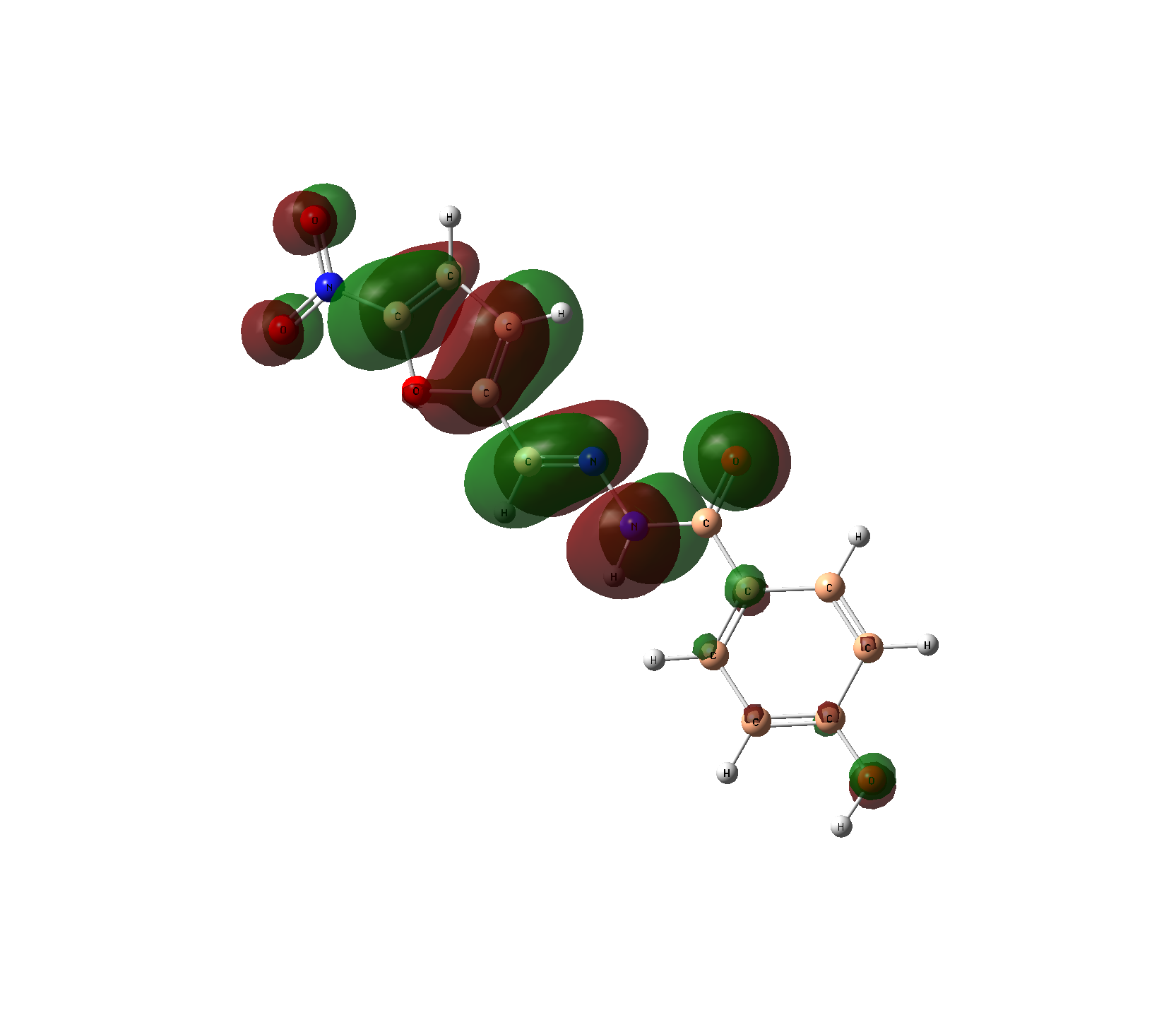 | → | 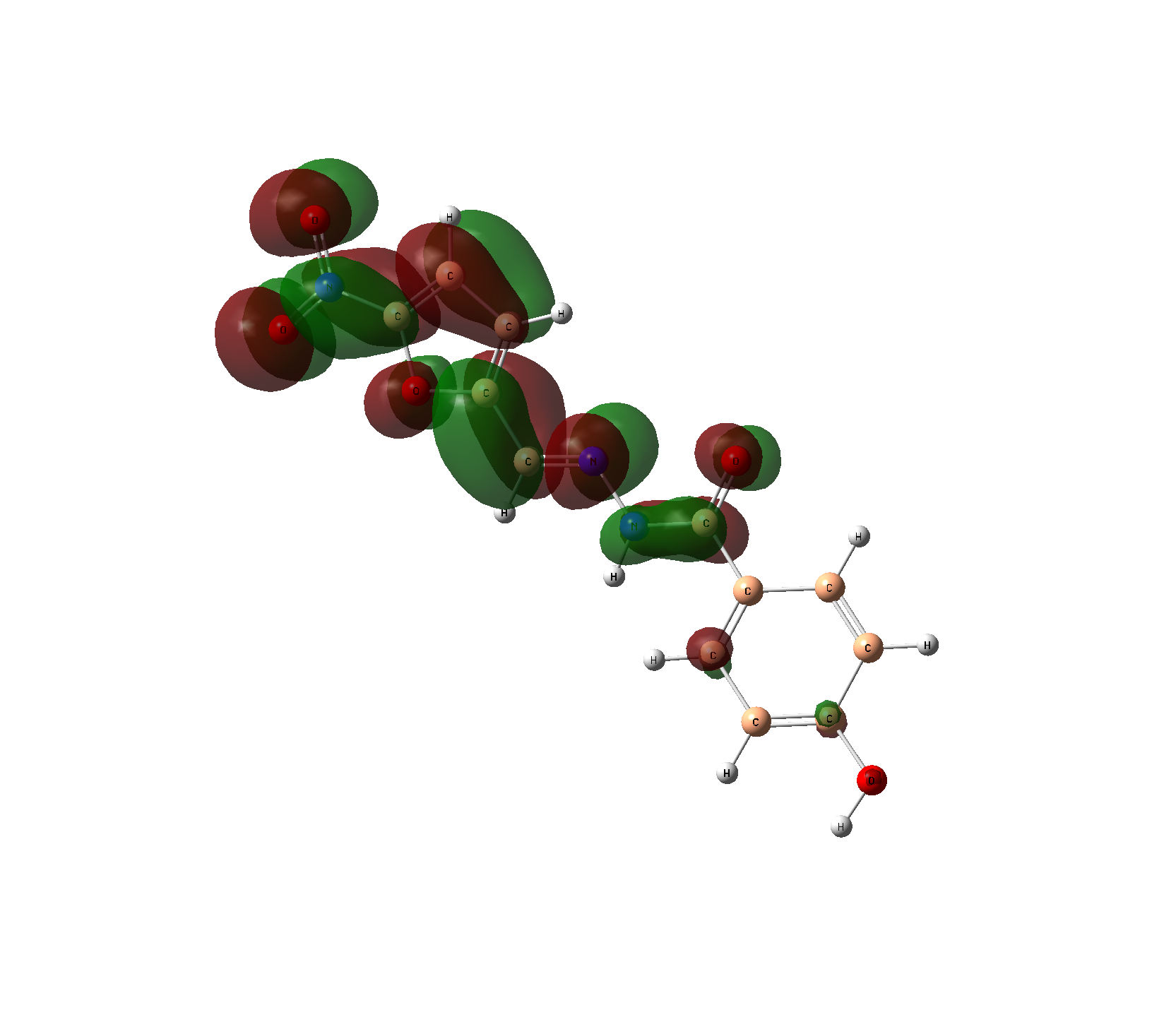 |
| AB | **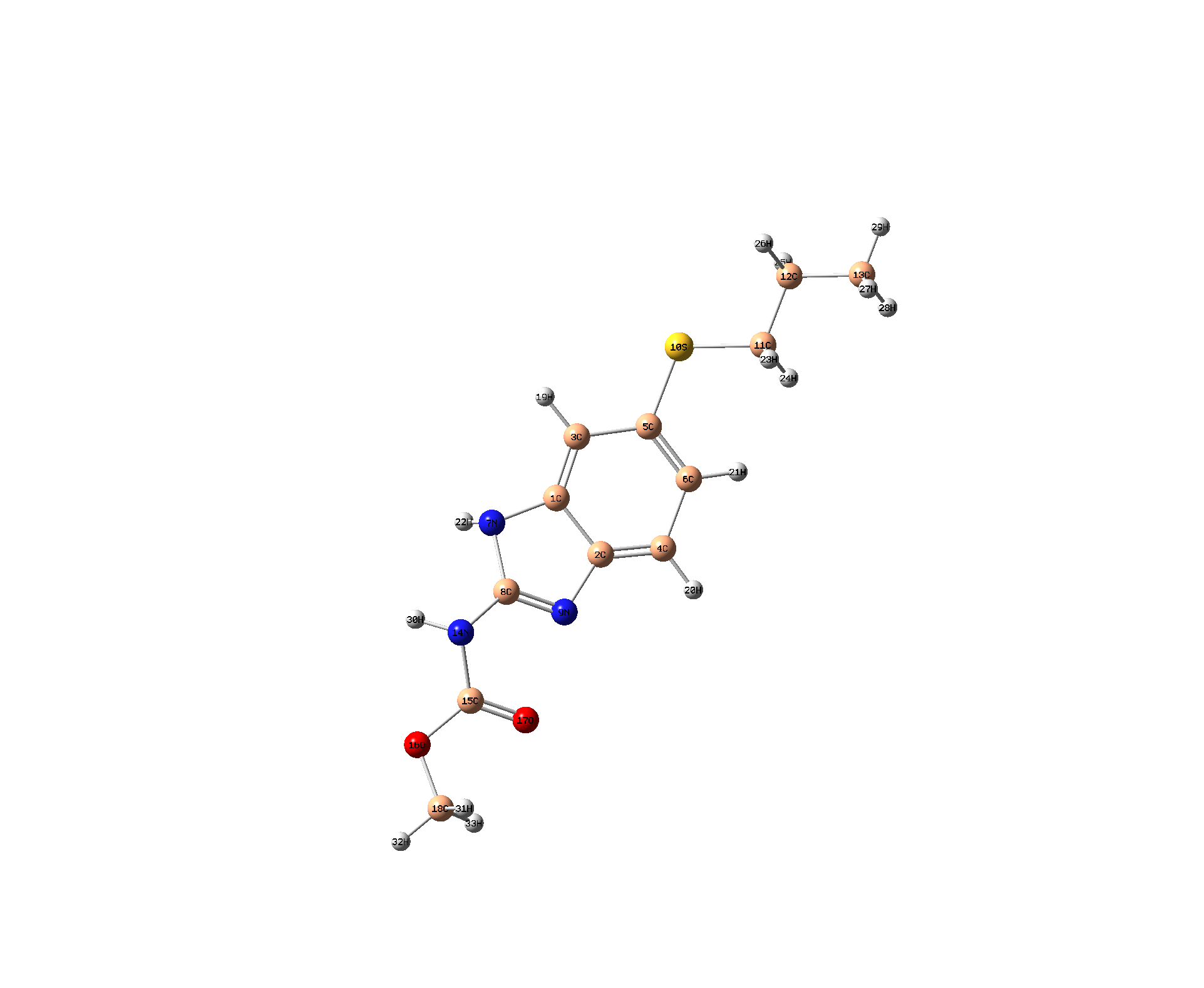** | 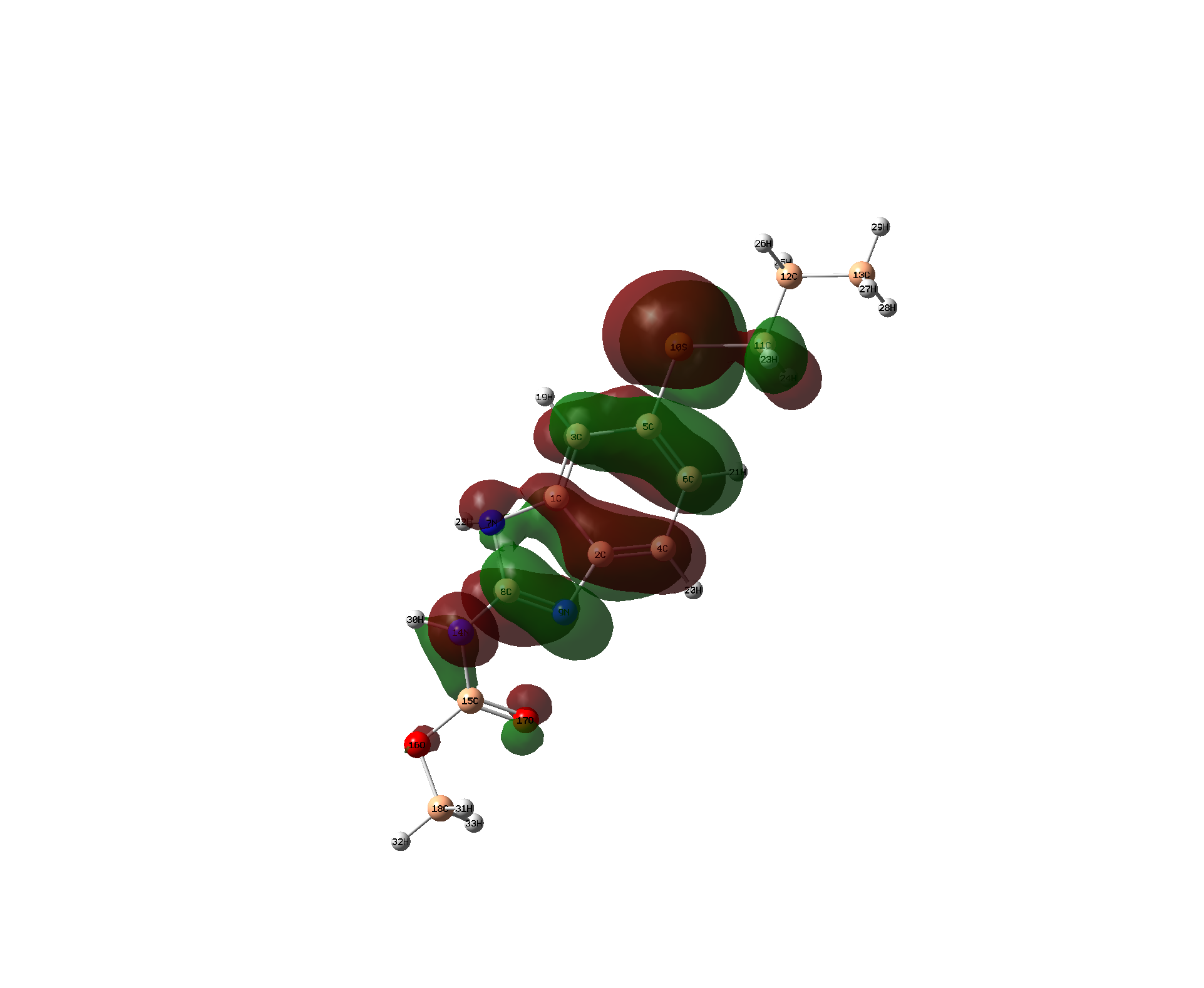 | → | 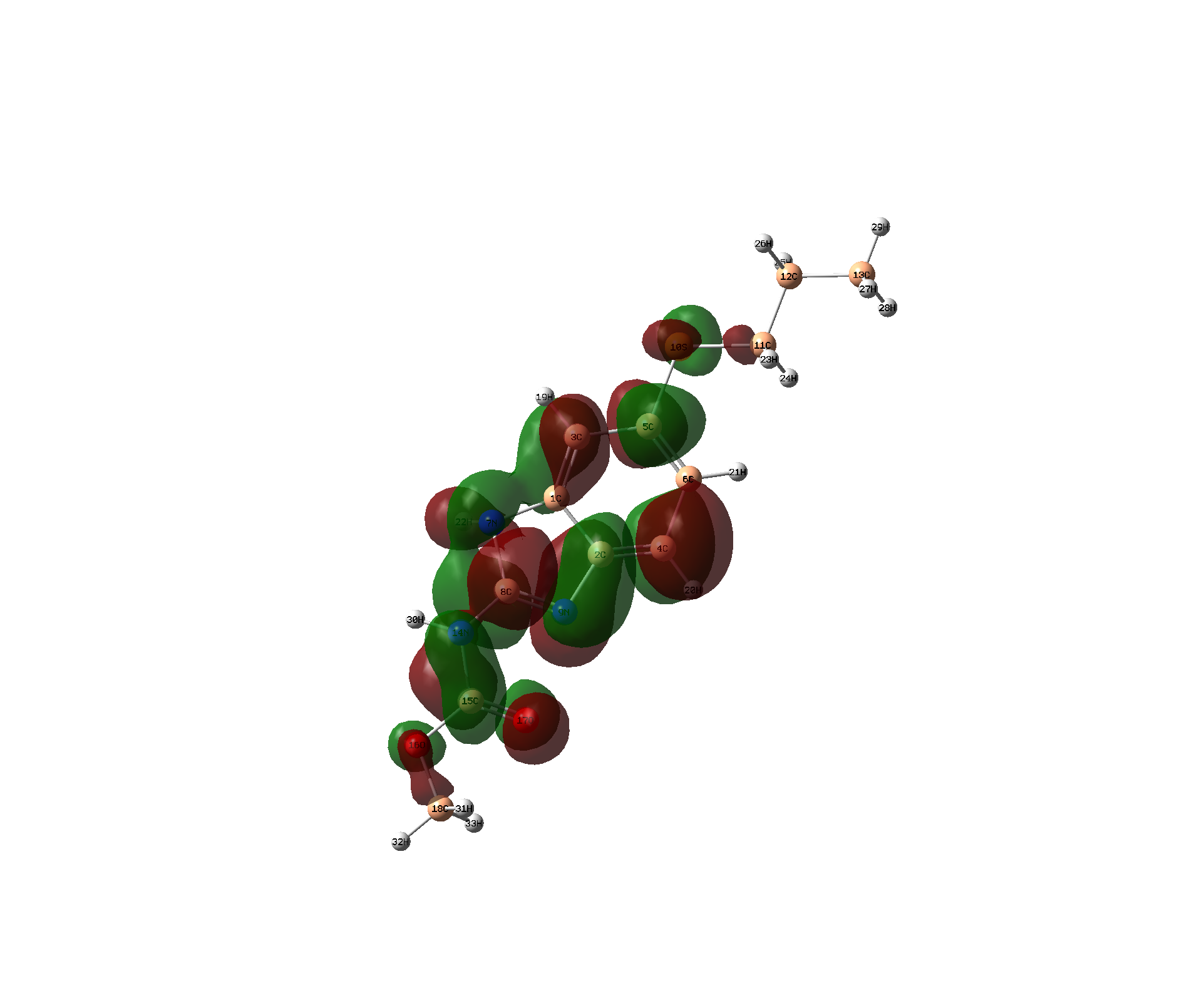 |
| Fig. (S.4): The 3D, HOMO, and LUMO of the Nifuroxazide (NF) and Albendazole (AB ligands | | | | |

| NF | | | | AB | | |
| --- | --- | --- | --- | --- | --- | --- |
| 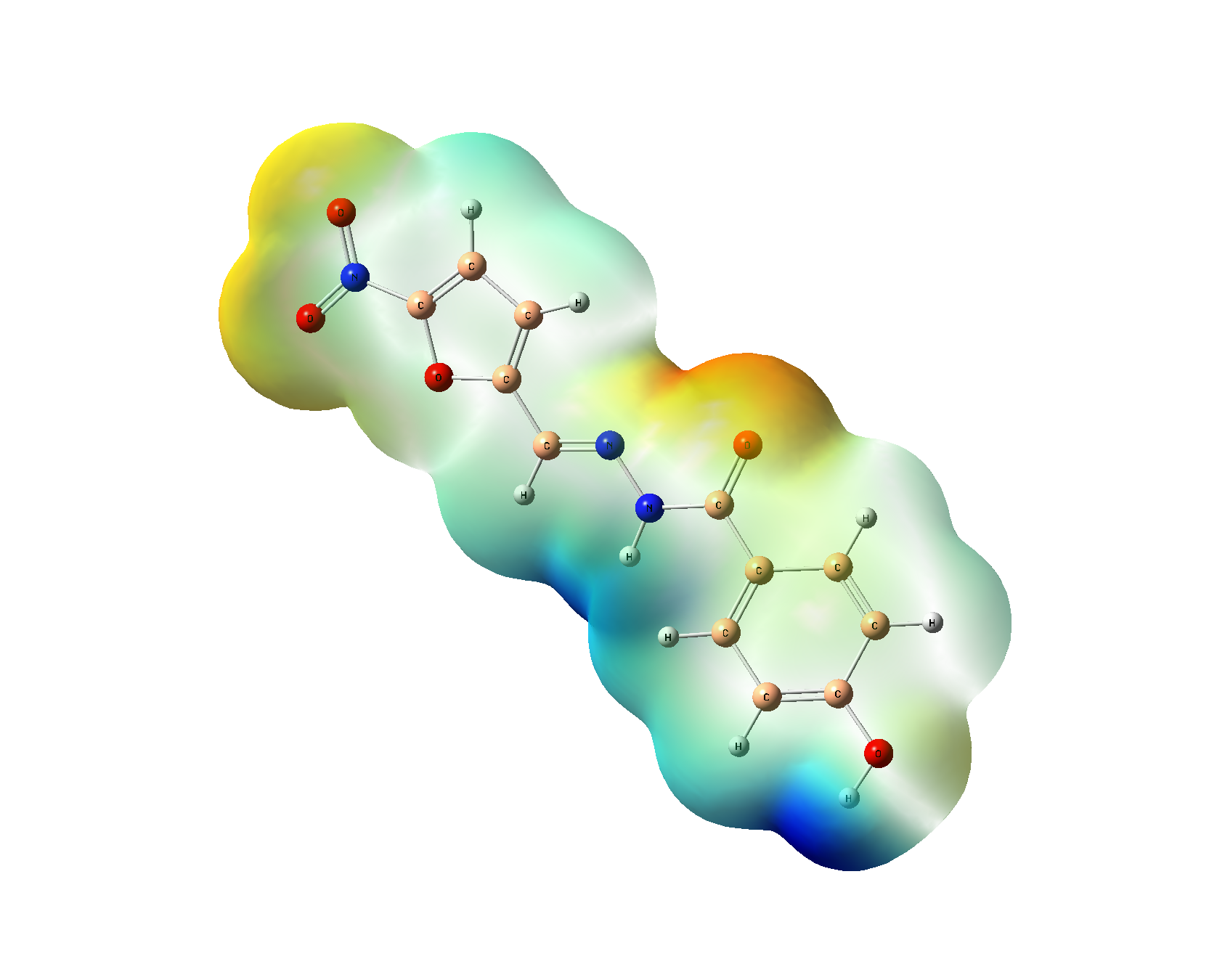 | | | | 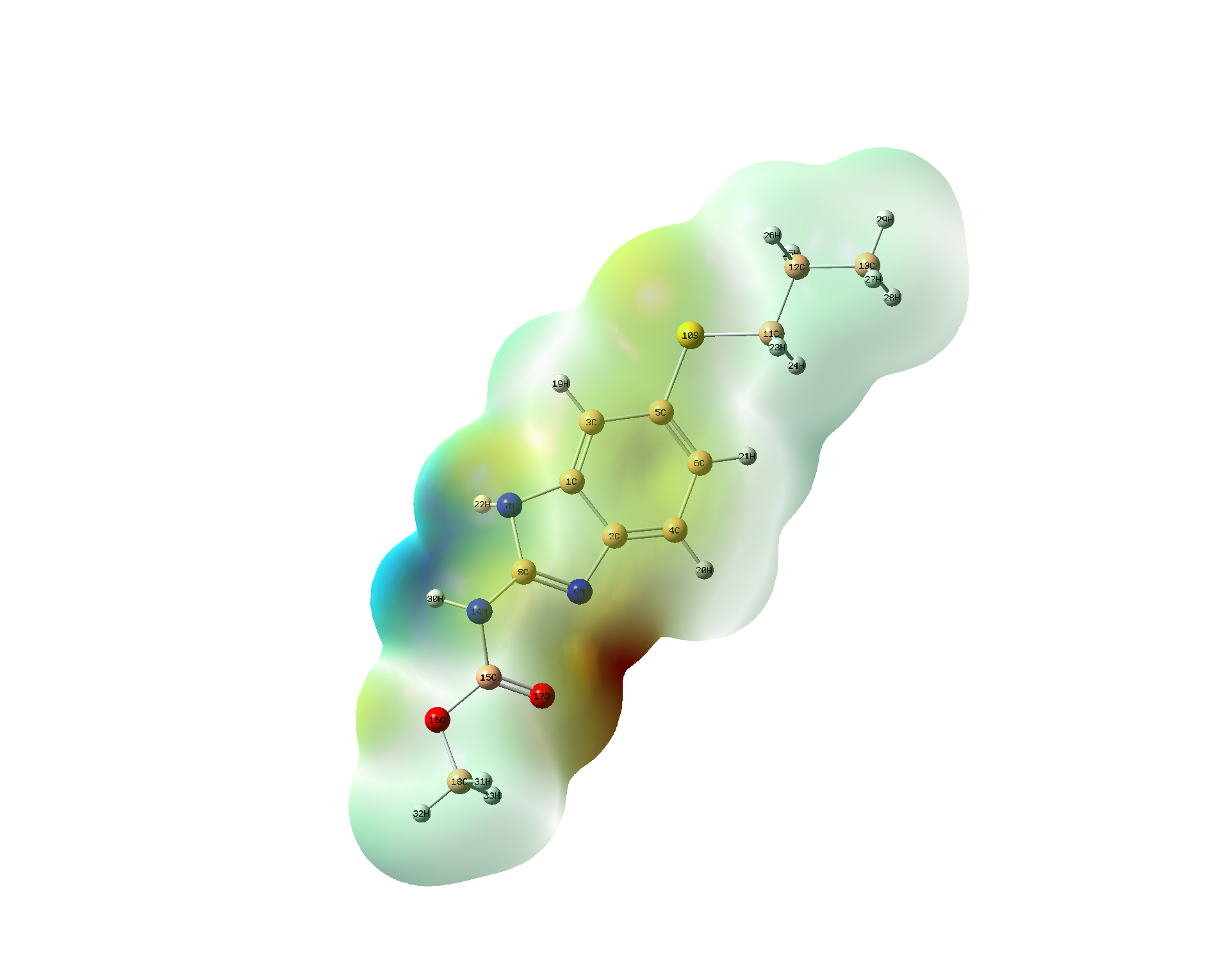 | | |
| -8.237x10^-2^ | | +8.237x10^-2^ | | -9.106x10^-2^ | +9.106x10^-2^ | |
| 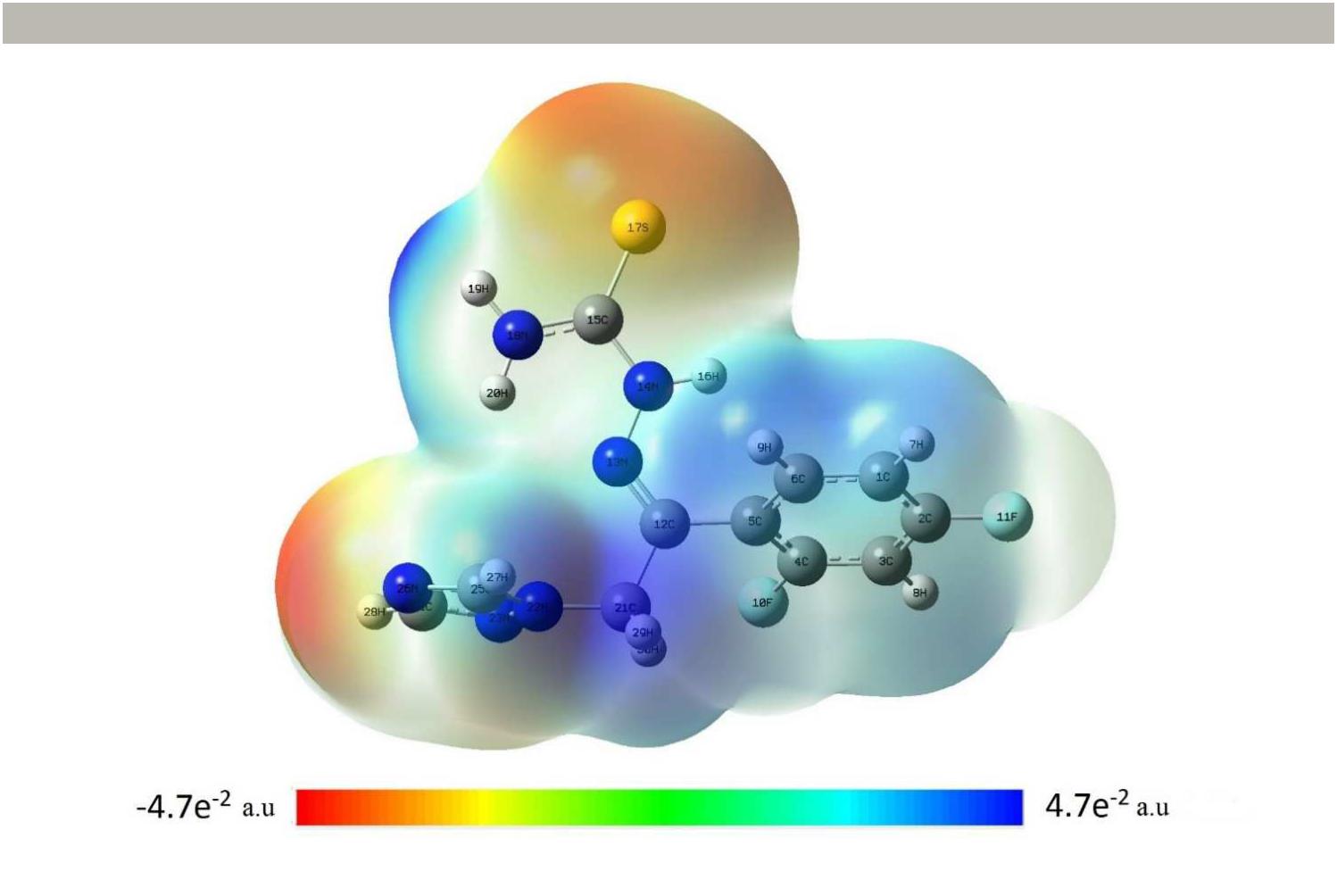 | | | | 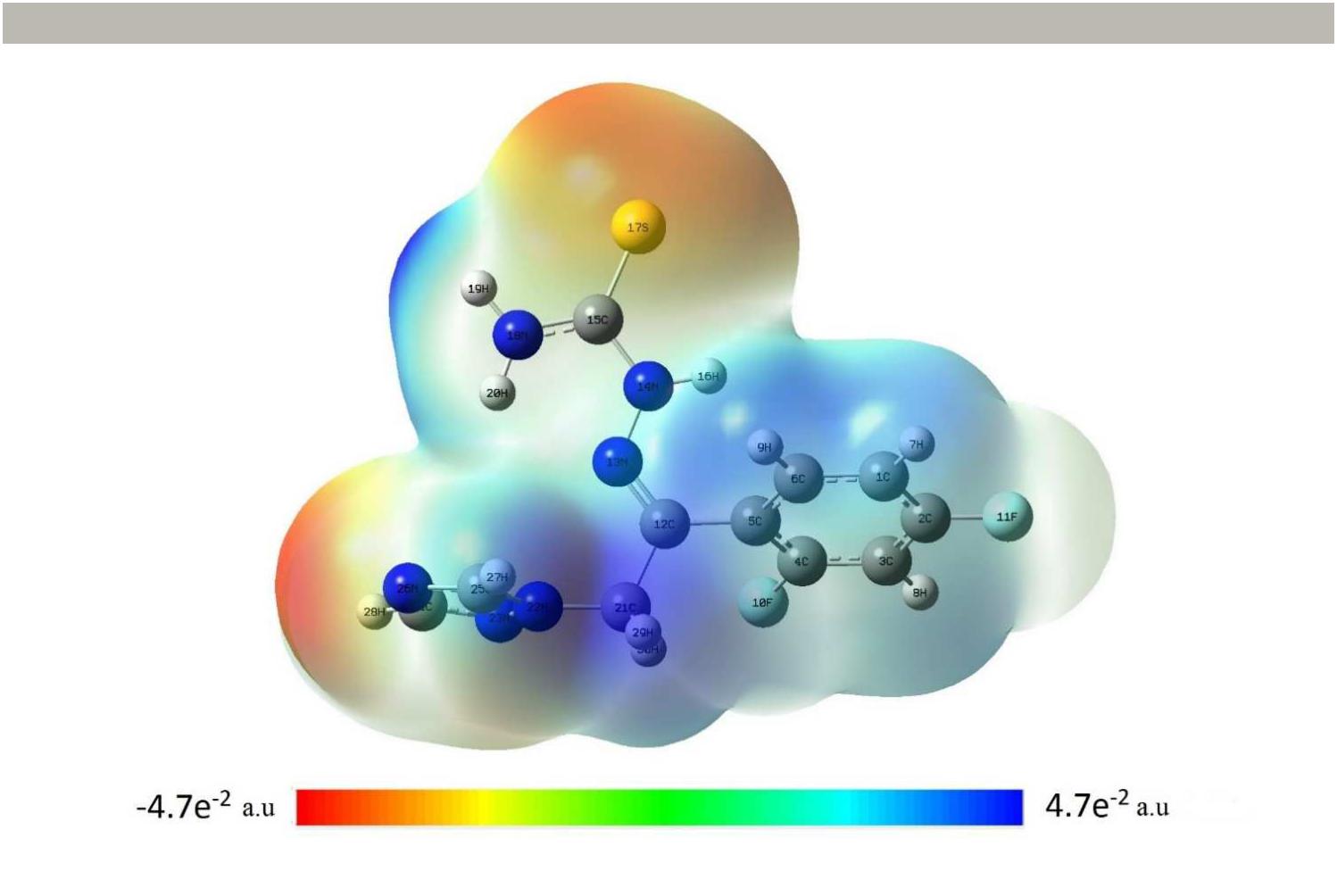 | | |
|  | | | |  |  | |
| FeABNF | | | CoABNF | | NiABNF | |
| 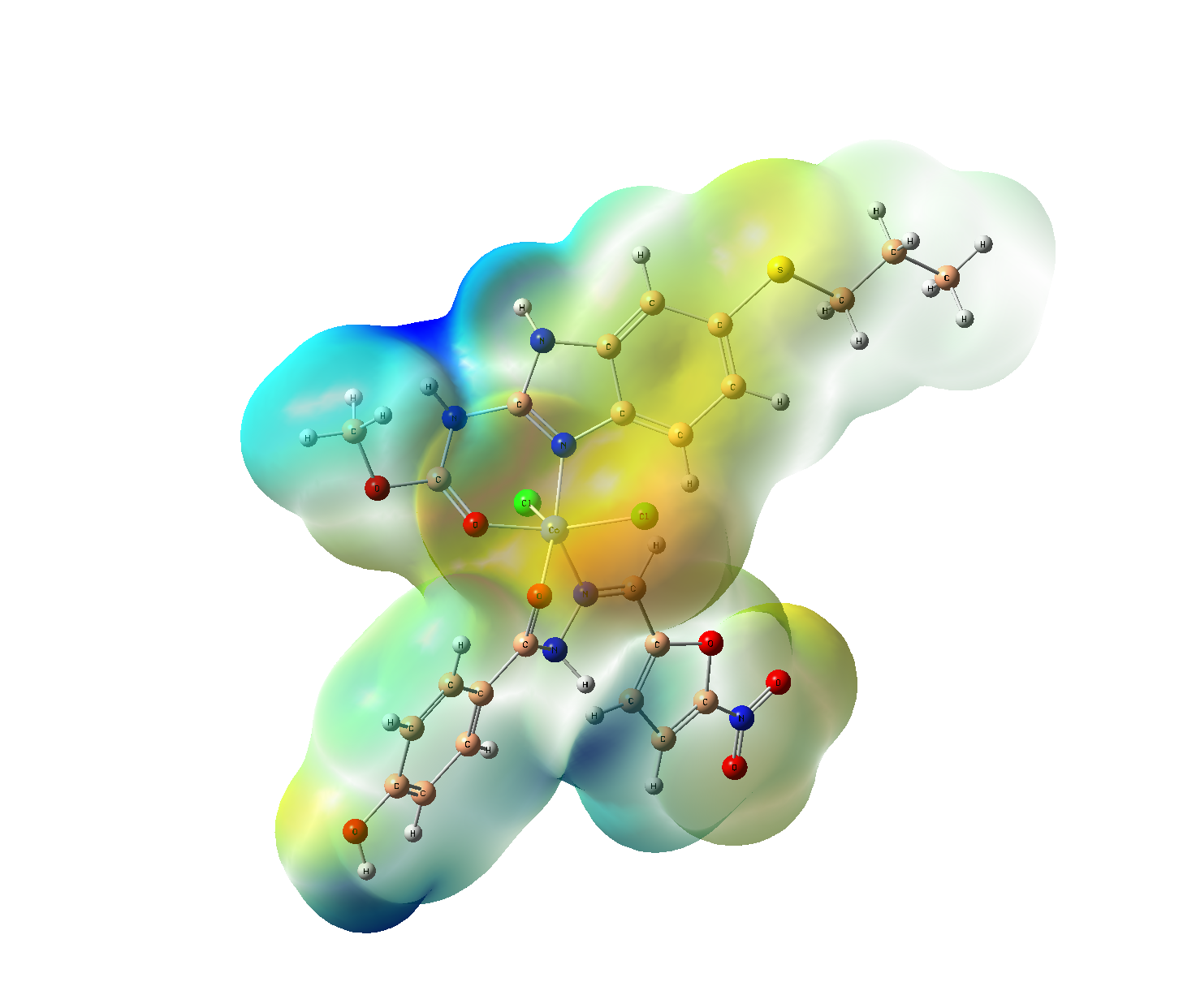 | | | **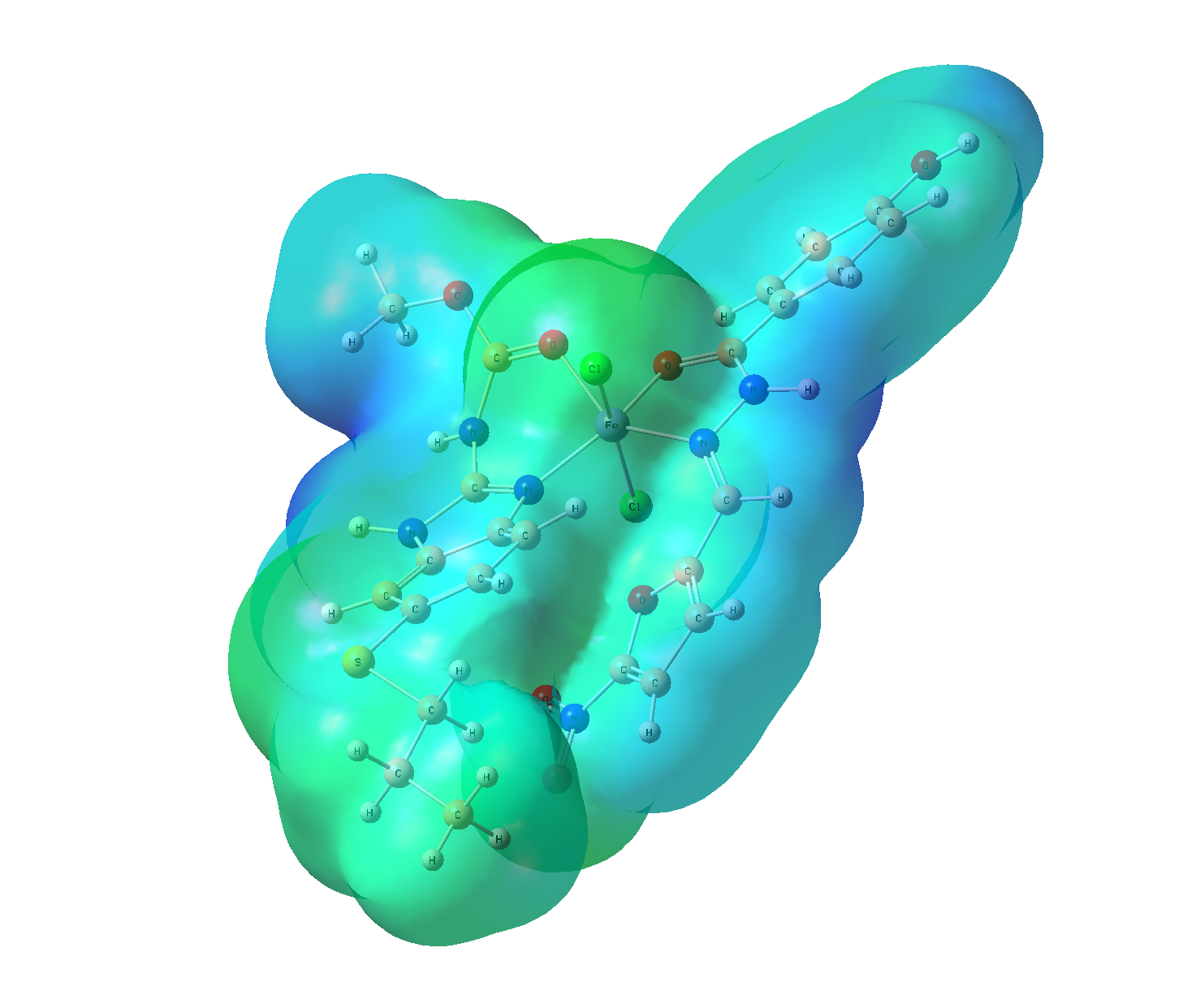** | | 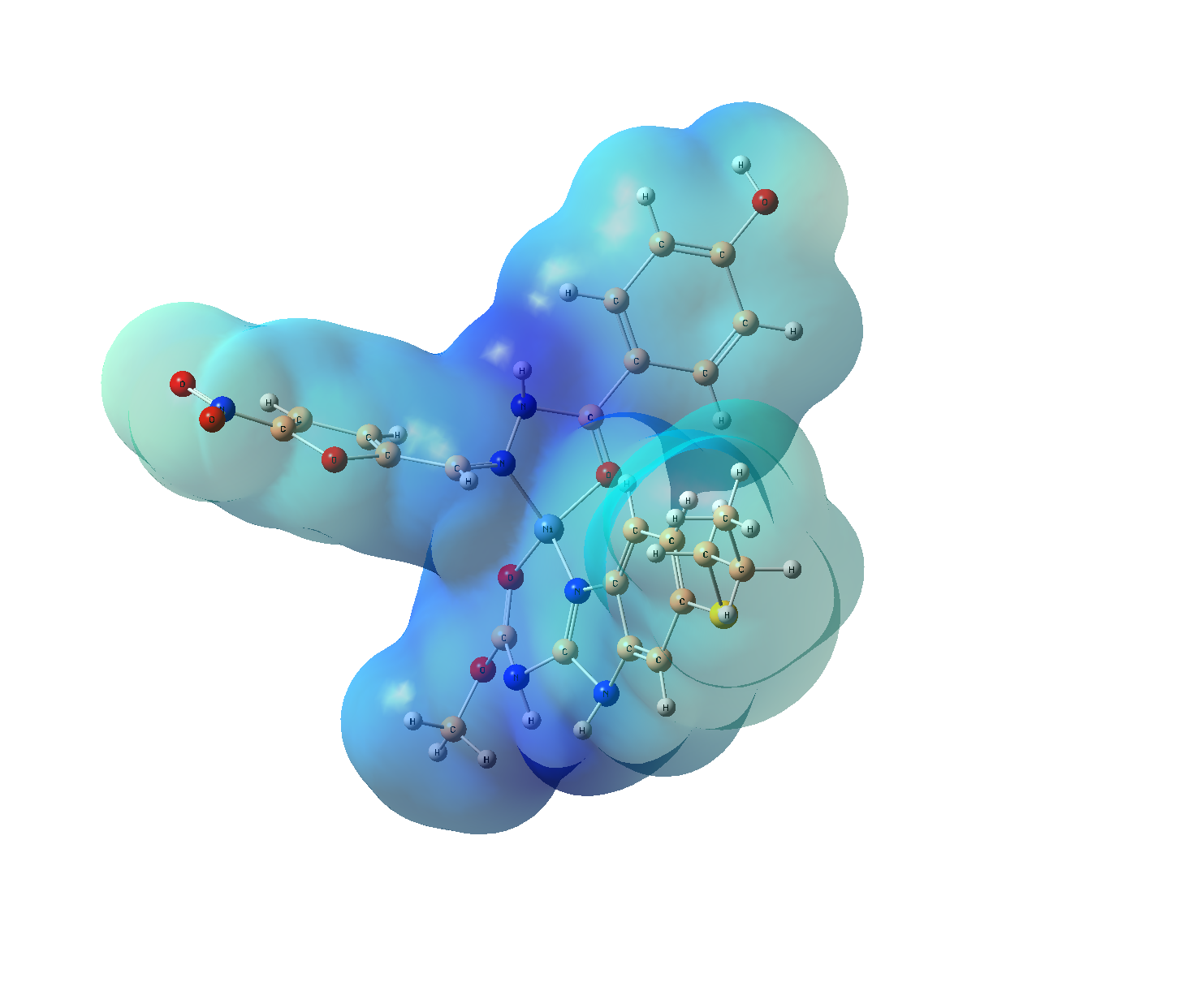 | |
| -9.030e-2 | 9.030e-2 | | -0.198e0 | 0.198e0 | -0.280e0 | 0.280e0 |
| 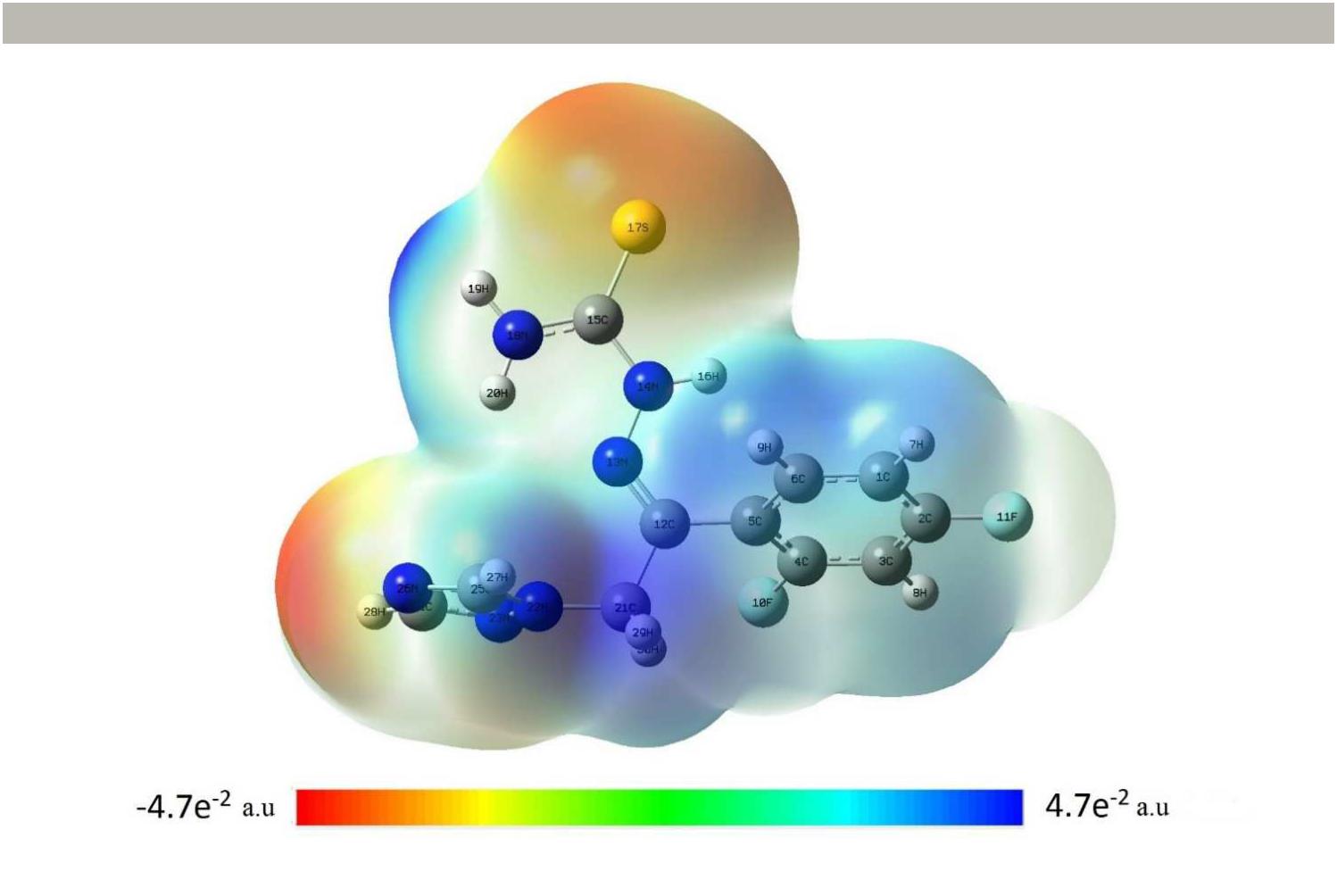 | | | 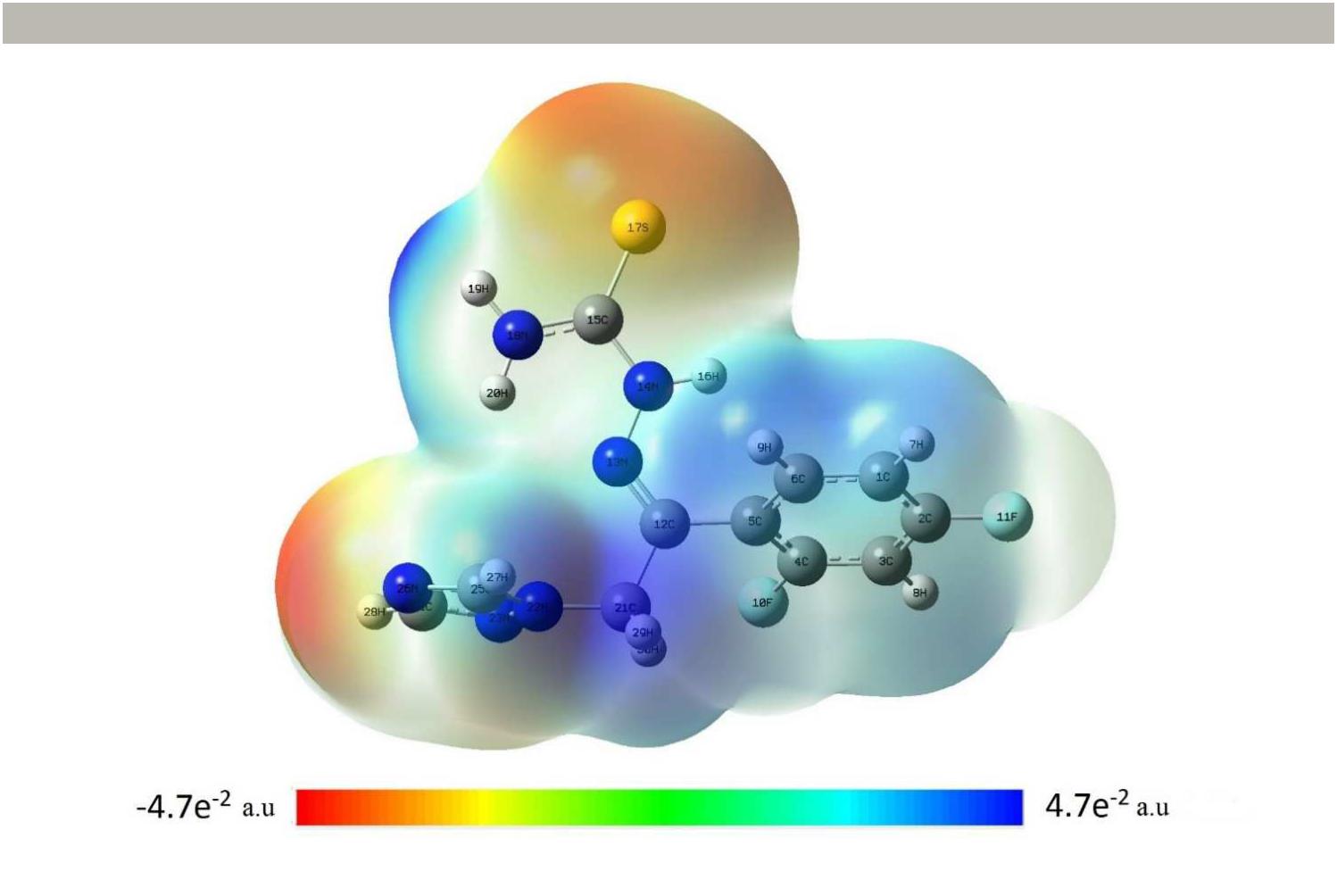 | | 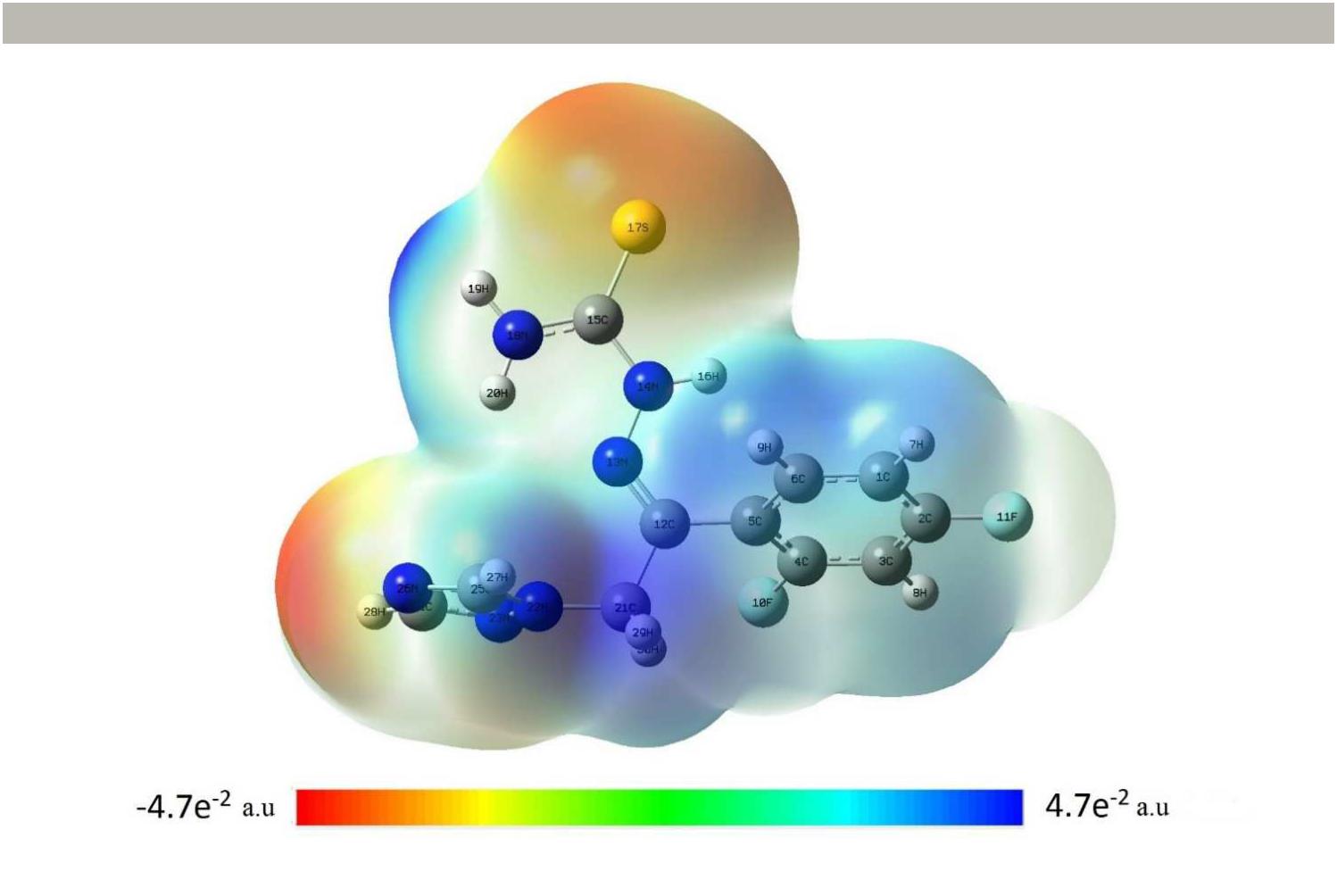 | |
| Fig. (S.5): The MEP of the Nifuroxazide (NF) and Albendazole (AB) ligands FeABNF, CoABNF, and NiABNF | | | | | | |

| (a) | 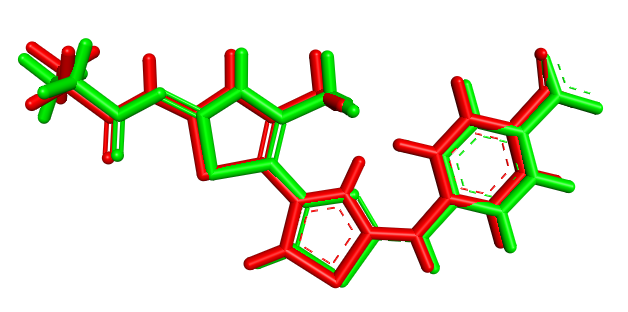 |
| --- | --- |
| (b) | 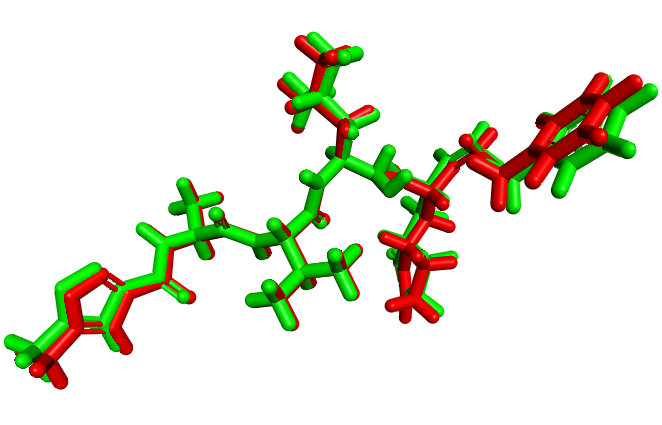 |
| Fig. (S.6): 3D representation of the superimposition of the co-crystallized (green) and the redocked (red) ligand of (a): PDB ID: 4DUH, and (b): PDB ID 6LU7. | |

| Table (S.1): Physical properties of the FeABNF, NiABNF, and CoABNF complexes | | | | | | |
| --- | --- | --- | --- | --- | --- | --- |
|  |  | **ligands** | | **Complexes** |  |  |
|  |  | **NF** | **AB** | FeABNF | NiABNF | CoABNF |
| Physical properties | Color | Yellowish |  | Pale-brown | light-green | reddish |
|  | Melting point (^o^C) | 282 | 208 | > 300 | > 300 | > 300 |
|  | Yield (%) | ---- | ---- | 80 | 80 | 80 |

| Table (S.2): conductivity of the FeABNF, NiABNF, and CoABNF complexes | | | | | | |
| --- | --- | --- | --- | --- | --- | --- |
|  |  | **ligands** | | **Complexes** |  |  |
|  |  | **NF** | **AB** | FeABNF | NiABNF | CoABNF |
| conductivity | µ_v_, Ω^−1^cm^2^mol^−1^ | ---- | ---- | 38.95 | 88.17 | 9.86 |
|  | assignment |  |  | 1:1 electrolyte | 1:2 electrolyte | Non-electrolyte |

| Table (S.3): UV-Vis. Spectra of the FeABNF, NiABNF, and CoABNF complexes | | | | | | |
| --- | --- | --- | --- | --- | --- | --- |
|  |  | **ligands** | | **Complexes** |  |  |
|  |  | **NF** | **AB** | **FeABNF** | **NiABNF** | **CoABNF** |
| UV-vis. | λ_max_, nm | 330 and 415 | 280 | 455 nm (21978.0219 cm^-1^) | 545 nm (18348.6238 cm^-1^) | 510 nm (19607.8431 cm^-1^) |
|  | |  |  | [3] | [4] | [5, 6] |

| Table (S.4): Effective magnetic moment of the FeABNF, NiABNF, and CoABNF complexes | | | | | | |
| --- | --- | --- | --- | --- | --- | --- |
|  |  | **ligands** | | **Complexes** |  |  |
|  |  | **NF** | **AB** | FeABNF | NiABNF | CoABNF |
| Effective magnetic moment | µ_eff_ (B.M) | ---- | ---- | 1.88 | 3.18 | 1.81 |

| Table (S.5): Antibacterial activity as diameter of zone inhibition in mm | | | | |
| --- | --- | --- | --- | --- |
|  | **Diameter of zone inhibition in mm** | | | |
|  | **Gram-positive bacteria** | | **Gram-negative bacteria** | |
|  | ***Bacillus Subtilis*** | ***Staph. Aureus*** | ***Escherichia coli*** | ***k. pneumonia*** |
| NF | 14 | 15 | 15 | 14 |
| AB | 13 | 14 | 14 | 14 |
| FeABNF | 27 | 26 | 26 | 28 |
| CoABNF | 30 | 27 | 28 | 29 |
| NiABNF | 30 | 28 | 28 | 29 |
| chloramphenicol | 33 | 31 | 30 | 31 |

| Table (S.6): Antibacterial activity as Activity index (%) | | | | |
| --- | --- | --- | --- | --- |
|  | **Activity index (%)** | | | |
|  | **Gram-positive bacteria** | | **Gram-negative bacteria** | |
|  | ***Bacillus Subtilis*** | ***Staph. Aureus*** | ***Escherichia coli*** | ***k. pneumonia*** |
| NF | 42.42 | 48.39 | 50.00 | 45.16 |
| AB | 39.39 | 45.16 | 46.67 | 45.16 |
| FeABNF | 81.82 | 83.87 | 86.67 | 90.32 |
| CoABNF | 90.91 | 87.10 | 93.33 | 93.55 |
| NiABNF | 90.91 | 90.32 | 93.33 | 93.55 |
| chloramphenicol | 100 | 100 | 100 | 100 |

| Table (S.7): Anti-fungal activity as diameter of zone inhibition in mm | | |
| --- | --- | --- |
|  | **Diameter of zone inhibition in mm** | |
| **Comp.** | ***Candida albicans*** | ***Aspergillus niger*** |
| NF | 11 | 10 |
| AB | 10 | 9 |
| FeABNF | 18 | 19 |
| CoABNF | 20 | 19 |
| NiABNF | 20 | 19 |
| clotrimazole | **23** | **22** |

| Table (S.8): Anti-fungal activity as Activity index (%) | | |
| --- | --- | --- |
|  | **Activity index (%)** | |
| **Comp.** | ***Candida albicans*** | ***Aspergillus niger*** |
| NF | 47.83 | 45.45 |
| AB | 0.00 | 0.00 |
| FeABNF | 78.26 | 86.36 |
| CoABNF | 86.96 | 86.36 |
| NiABNF | 86.96 | 86.36 |
| clotrimazole | 100 | 100 |

| Table (S.9): Anti-inflammatory results as Mean percentage inhibition, and IC_50_ of the studied compounds | | | | | |
| --- | --- | --- | --- | --- | --- |
|  | % inhibition ± SD | | | | |
|  | AB | NF | FeABNF | CoABNF | NiABNF |
| 12.5 | 14.00 | 6.00 | 10.00 | 14.00 | 16.00 |
| 25 | 27.00 | 18.00 | 23.00 | 30.00 | 34.00 |
| 50 | 44.00 | 32.00 | 41.00 | 48.00 | 52.00 |
| 100 | 66.00 | 54.00 | 64.00 | 72.00 | 76.00 |
| 200 | 88.00 | 74.00 | 83.00 | 90.00 | 93.00 |
| IC_50_ | 114.86 | 106.74 | 72.87 | 64.98 | 56.97 |

| Table (S.10): molecular docking data of the AB, NF, and their FeABNF, CoABNF, and NiABNF complexes against 4DUH receptor | | | | |
| --- | --- | --- | --- | --- |
|  | Amino acid residue | distance | Interaction type | Binding energy (kcal/mol) |
| NF | GLY117 | 4.93 | Electrostatic | -7.10 |
|  | GLU42 | 3.91 | Electrostatic |  |
|  | ASP73 | 2.20 | Hydrogen Bond |  |
|  | ALA100 | 2.17 | Hydrogen Bond |  |
|  | LYS103 | 4.94 | Electrostatic |  |
|  | LYS103 | 3.91 | Hydrophobic |  |
|  | ILE78 | 4.44 | Hydrophobic |  |
| AB | GLU50 | 3.99 | Electrostatic | -6.90 |
|  | GLU50 | 2.77 | Hydrogen Bond |  |
|  | GLY77 | 3.00 | Hydrogen Bond |  |
|  | LYS103 | 4.23 | Electrostatic |  |
|  | GLU50 | 4.31 | Electrostatic |  |
|  | PRO79 | 4.58 | Hydrophobic |  |
|  | VAL43 | 4.60 | Hydrophobic |  |
|  | VAL120 | 4.78 | Hydrophobic |  |
|  | VAL167 | 4.52 | Hydrophobic |  |
|  | ILE78 | 4.59 | Hydrophobic |  |
| FeABNF | LYS10 | 4.23 | Electrostatic | -8.10 |
|  | GLU50 | 4.03 | Electrostatic |  |
|  | ILE78 | 5.37 | Hydrophobic |  |
|  | PRO79 | 4.87 | Hydrophobic |  |
| CoABNF | ARG76 | 4.84 | Electrostatic | -8.60 |
|  | LYS103 | 3.90 | Electrostatic |  |
|  | ASP49 | 3.45 | Electrostatic |  |
|  | GLY101 | 2.16 | Hydrogen Bond |  |
|  | ARG76 | 3.46 | Hydrogen Bond |  |
|  | LYS103 | 4.82 | Electrostatic |  |
|  | ARG136 | 4.09 | Electrostatic |  |
|  | ALA53 | 4.18 | Hydrophobic |  |
|  | LYS103 | 4.56 | Hydrophobic |  |
|  | ILE78 | 5.31 | Hydrophobic |  |
|  | ILE94 | 4.37 | Hydrophobic |  |
|  | LYS103 | 4.31 | Hydrophobic |  |
| NiABNF | ARG76 | 5.36 | Electrostatic | -8.90 |
|  | LYS103 | 5.45 | Electrostatic |  |
|  | ARG136 | 5.41 | Electrostatic |  |
|  | GLU50 | 3.79 | Electrostatic |  |
|  | ARG76 | 3.02 | Hydrogen Bond |  |
|  | GLY77 | 2.63 | Hydrogen Bond |  |
|  | ARG76 | 3.81 | Electrostatic |  |
|  | LYS103 | 4.45 | Electrostatic |  |
|  | GLU50 | 3.92 | Electrostatic |  |
|  | ARG76 | 5.47 | Hydrophobic |  |
|  | PRO79 | 4.67 | Hydrophobic |  |
|  | ALA90 | 5.11 | Hydrophobic |  |

| Table (S.11): molecular docking data of the AB, NF, and their FeABNF, CoABNF, and NiABNF complexes against 6LU7 receptor | | | | |
| --- | --- | --- | --- | --- |
|  | Amino acid residue | distance | Interaction type | Binding energy (kcal/mol) |
| AB | CYS145 | 3.47772 | Hydrogen Bond | -6.40 |
|  | ASN142 | 2.45372 | Hydrogen Bond |  |
|  | GLU166 | 2.80954 | Hydrogen Bond |  |
|  | GLY143 | 2.88353 | Hydrogen Bond |  |
|  | HIS41 | 5.92131 | Hydrophobic |  |
|  | HIS163 | 4.62502 | Hydrophobic |  |
|  | HIS172 | 4.71225 | Hydrophobic |  |
|  | CYS145 | 4.653 | Hydrophobic |  |
| NF | GLY143 | 2.62154 | Hydrogen Bond | -6.80 |
|  | SER144 | 2.57973 | Hydrogen Bond |  |
|  | CYS145 | 2.10452 | Hydrogen Bond |  |
|  | GLU166 | 2.53093 | Hydrogen Bond |  |
| FeABNF | HIS41 | 5.06767 | Electrostatic | -8.70 |
|  | HIS41 | 2.93051 | Hydrogen Bond |  |
|  | HIS41 | 2.90418 | Hydrogen Bond |  |
|  | ASN142 | 2.20696 | Hydrogen Bond |  |
|  | MET49 | 3.01358 | Hydrophobic |  |
| CoABNF | HIS172 | 5.3726 | Electrostatic | -9.10 |
|  | GLU166 | 2.92735 | Electrostatic |  |
|  | ASN142 | 2.92111 | Hydrogen Bond |  |
|  | HIS172 | 2.46154 | Hydrogen Bond |  |
| NiABNF | GLU166 | 5.1458 | Electrostatic | -9.40 |
|  | HIS163 | 2.35339 | Hydrogen Bond |  |
|  | LEU141 | 1.86695 | Hydrogen Bond |  |
|  | ASN142 | 2.70195 | Hydrogen Bond |  |
|  | GLU166 | 1.99332 | Hydrogen Bond |  |
|  | PHE140 | 1.53913 | Hydrogen Bond |  |
|  | LEU141 | 4.74209 | Hydrophobic |  |

**Referances**

[1] A. Gahlan, M. El-Mottaleb, N. Badawy, F. Kamale, S.H. Ali, Spectrophotometeric studies on binary and ternary complexes of some metal ions with alizarin red S and cysteine, Int. J. Adv. Res 2(10) (2014) 570-584.

[2] R.M. El-Khatib, L.A.-M.E. Nassr, Spectrophotometric study of some Mn (II) ternary complexes and their analytical applications, Monatshefte für Chemie-Chemical Monthly 140 (2009) 1139-1142.

[3] G.A. Al‐Hazmi, K.S. Abou‐Melha, N.M. El‐Metwaly, I. Althagafi, F. Shaaban, R. Zaky, Green synthesis approach for Fe (III), Cu (II), Zn (II) and Ni (II)‐Schiff base complexes, spectral, conformational, MOE‐docking and biological studies, Applied Organometallic Chemistry 34(3) (2020) e5403.

[4] H. Temel, Ü. Çakir, H.İ. Uğraş, M. Şekerci, The synthesis, characterization and conductance studies of new Cu (II), Ni (II) and Zn (II) complexes with the Schiff base derived from 1, 2-bis-(o-aminophenoxy) ethane and salicylaldehyde, Journal of Coordination Chemistry 56(11) (2003) 943-951.

[5] N.G. Yernale, M.B.H. Mathada, Preparation of octahedral Cu (II), Co (II), Ni (II) and Zn (II) complexes derived from 8-formyl-7-hydroxy-4-methylcoumarin: Synthesis, characterization and biological study, Journal of Molecular Structure 1220 (2020) 128659.

[6] R. Rai, Metal complexes of 5-(o) hydroxyphenyl-1, 3, 4-oxadiazole-2-thione, Journal of Inorganic and Nuclear Chemistry 42(3) (1980) 450-453.
